# Multifunctional Edged‐Satellite AuAg Nanoparticles‐Based Integration Platform for Screening, Validation, and Elimination of *Vibrio* Bacteria

**DOI:** 10.1002/advs.202516240

**Published:** 2025-11-05

**Authors:** Yixuan Wu, Jiadong Chen, Liyan Bi, Zhiyang Zhang, Xiaoyan Wang, Longwen Fu, Qian Yang, Jaebum Choo, Lingxin Chen

**Affiliations:** ^1^ CAS Key Laboratory of Coastal Environmental Processes and Ecological Remediation Yantai Institute of Coastal Zone Research Chinese Academy of Sciences Yantai 264003 China; ^2^ Department of Chemistry Chung‐Ang University Seoul 06974 South Korea; ^3^ School of Special Education School of Pharmacy Binzhou Medical University Yantai 264003 China; ^4^ The Institute for Advanced Study Shaoxing University Shaoxing 312000 China

**Keywords:** antimicrobials, multifunctional nanoparticles, peroxidase‐like catalytic, surface‐enhanced Raman scattering, vibrio bacteria

## Abstract

Multifunctional materials converge various physical and chemical properties, while often being hampered by complicated synthesis procedures and inconsistent performance. A straightforward strategy is presented for synthesizing robust multifunctional plasmonic nanoparticles (NPs), AuAgNPs with edged satellites (ES‐AuAgNPs). The ES‐AuAgNPs possess various characteristics of i) alloyed Ag/Au with a 7:3 ratio, ii) numerous satellited NPs with narrow nanogaps on face‐to‐face edges, and iii) a well‐accessible surface deposited with antimicrobial components. These properties endow ES‐AuAgNPs with improved peroxidase (POD)‐like catalytic, surface‐enhanced Raman scattering (SERS), and antibacterial activities. By utilizing the triple‐performance of ES‐AuAgNPs and a modified specific aptamer probe, a portable, modular, and centimeter‐sized platform is designed for simultaneously screening, validating, and eliminating pandemic marine *Vibrio* bacteria: *parahaemolyticus*, *vulnificus*, and *alginolyticus*. Wherein, POD‐like catalytic‐based colorimetric capacity can rapidly screen the presence of these bacteria through visible color change, and SERS‐based detection validates bacterial identity with multiplexing capability. Meanwhile, the inherent antibacterial properties of ES‐AuAgNPs ensure the effective elimination of detected bacteria. Remarkable performance is achieved in rapid screening (2 min), sensitive validation (limit of detection, ≈50 CFU per mL), and efficient antimicrobility (45 min, 100%). The ES‐AuAgNPs constructed platform is believed to improve aquaculture management by seamlessly analyzing and eliminating bacteria.

## Graphical abstract

A simple method is developed to synthesize multifunctional nanoparticles (NPs), AuAgNPs with edged satellites (ES‐AuAgNPs). These NPs demonstrate peroxidase‐like (POD‐like) activity, SERS ability, and antibacterial properties. Utilizing these multifunctional capabilities, a portable, syringe‐sized biosensor is created that integrates screening, identification and elimination of *Vibrio* species within a single platform.

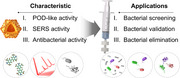

## 1 Introduction

A versatile platform constructed using multifunctional nanomaterials presents a promising technology capable of seamlessly integrating various applications in diverse fields, particularly for sensing and processing infectious pathogens.^[^ ^1^, ^2^ ^]^ It offers a streamlined workflow and enhanced analytical performance as a result of its capability to simultaneously screen‐specific bacteria, identify their species, and conduct sterilization procedures while consuming minimal resources.^[^ ^3^, ^4^ ^]^ Conventional methods, by contrast, necessitate laboratory‐scale space, expert operations, and rigorous pre‐/post‐processing procedures for the same contents.^[^ ^5^ ^]^ Despite its potential, realizing such an ideal platform remains challenging due to the fundamental lack of multifunctional nanomaterials with a balanced performance.^[^ ^6^ ^]^

Plasmonic nanoparticles (NPs) such as gold (Au), silver (Ag), and platinum (Pt) are the most promising candidates, given their various and tunable properties.^[^ ^7^ ^]^ Among these multifaceted performances, the enzyme‐mimicking catalytic behaviors allow them to serve as nanozymes, facilitating the rapid screening of bacteria through colorimetric assays.^[^ ^8^ ^]^ This property provides an on‐site detection strategy to determine the presence of bacteria, while the colorimetric‐based assay alone cannot sensitively distinguish multiple strains, and this challenge is exacerbated by the host's risk of various bacterial infections.^[^ ^9^ ^]^ An effective way to address this limitation is to leverage their other property, surface‐enhanced Raman scattering (SERS), known for its multiplexing capability and single‐molecular detection sensitivity.^[^ ^10^ ^]^ Thus, the synergy of colorimetric‐rapid detection and SERS‐sensitive validation has emerged as one of the most popular precision detection technologies.^[^ ^11^ ^]^ Additionally, the versatility of plasmonic NPs endows sensing platforms with functions beyond mere detection and analysis. By releasing metal ions and/or reactive oxygen species (ROS) to disrupt biofilms and eventually achieve sterilization.^[^ ^12^ ^]^ Integrating and leveraging multiple aforementioned functionalities, rather than relying on a single one, can yield substantial benefits. This comprehensive approach provides an all‐encompassing on‐site analysis strategy, encompassing both the detection and sterilization of pathogens.

AgNPs typically serve as multifunctional NPs with enzyme‐like catalytic, SERS, and antibacterial activities.^[^ ^13^ ^]^ The enhancement of each aspect can be achieved through three primary methods: alloying with hetero elements, creating nanogap structures, and optimizing accessible surface properties. Alloying treatment could alter the electronic property, enhance hot carrier utilization, and ultimately improve catalytic efficiency, which is the primary method used to enhance its enzyme‐like catalytic performance.^[^ ^14^ ^]^ Designing nanogap (or tip/edge‐shaped) structures is the most effective way to improve SERS performance. It can produce electromagnetic hotspots caused by localized surface plasmon resonance (LSPR), thus improving their SERS detection performance.^[^ ^15^ ^]^ In addition, an accessible surface coupled with a stable chemical/physical ability enhances microbial affinity and ensures sustained antibacterial effectiveness.^[^ ^16^ ^]^ However, concurrently improving all three properties is highly challenging as it involves fine engineering at the nanoscale. And, various parameters, reducing agents, etching/deposition processes, and ligand exchange, should be optimized to balance the performance of each aspect. Though some of the bi/tri‐metallic NPs have been developed, most of them have only improved two of the desired performances, which fail to realize the screening, classification, and elimination of bacteria simultaneously.^[^ ^17^, ^18^ ^]^

In this work, we employed AgNPs as a template and introduced a facile method to achieve alloying, nanogap creation, and an accessible surface. We synthesized ES‐AuAgNPs that simultaneously exhibit high‐performance peroxidase‐like (POD) catalytic activity, SERS ability, and antimicrobial properties. These NPs were then utilized to construct a versatile platform for the screening, validating, and removing of three pandemic marine *Vibrios*: *Vibrio parahaemolyticus*, *Vibrio vulnificus*, and *Vibrio alginolyticus*.

## 2 Results and Discussion

### 2.1 Synthesis Principle of ES‐AuAgNPs

**Figure 1:**
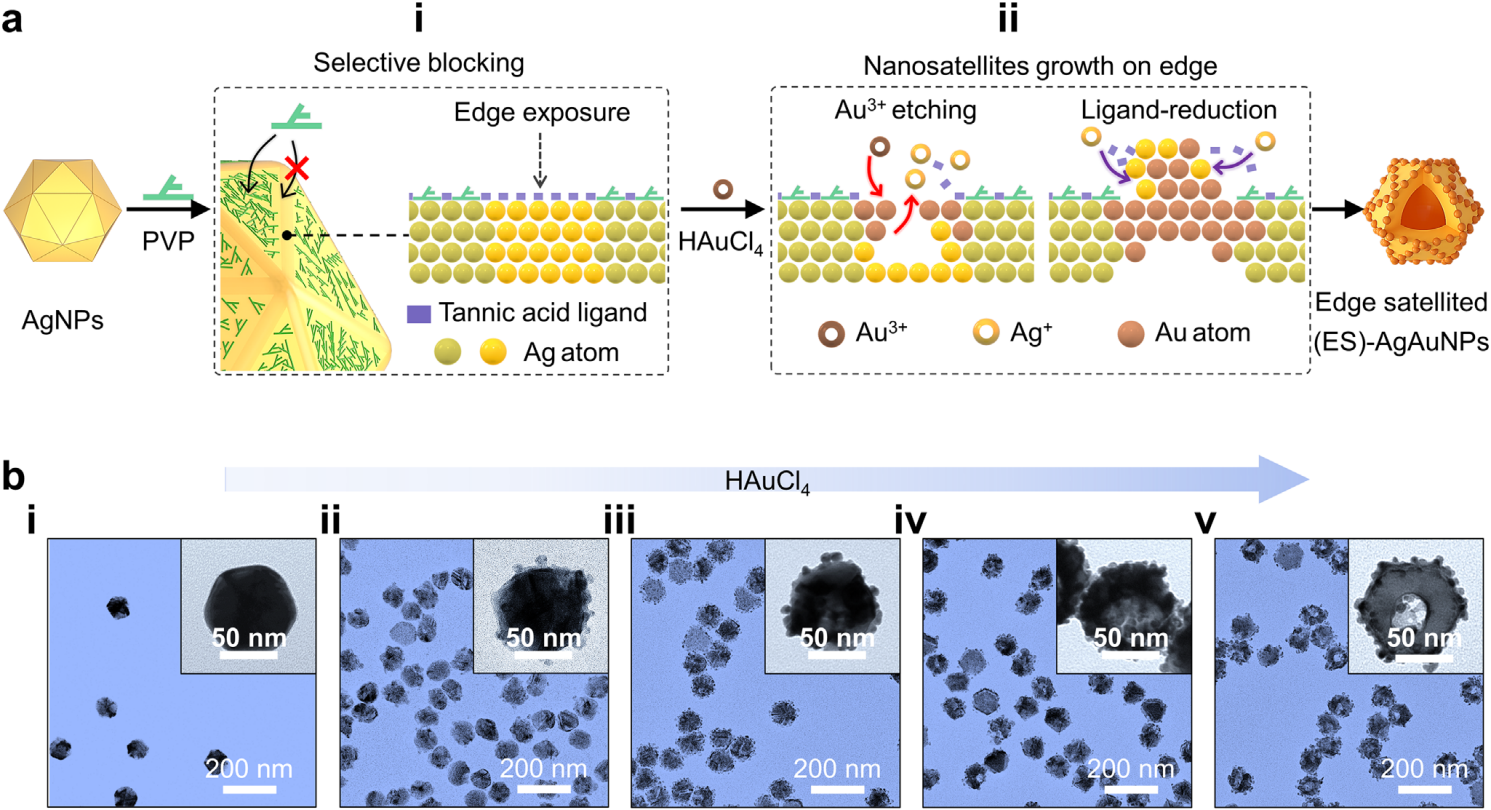
a) Principle of ES‐AuAgNPs synthetization: (i) PVP selectively blocking, (ii) HAuCl_4_ and tannic acid ligand‐induced nanosatellite formation. b) TEM images of ES‐AuAgNPs fabricated with various HAuCl_4_ concentrations: (i) 0, (ii) 5, (iii) 10, (iv) 15, and (v) 20 mm.

**Figure** **1**a illustrates the principle of ES‐AuAgNPs synthetization, and it merely entails the successive addition of PVP and HAuCl_4_ to a solution containing tannic acid‐capped AgNPs. Wherein, PVP, a typical blocking agent, exhibits a stronger affinity for the flat faces of AgNPs composed of {111} facets over the edges between these flat faces (Figure S1, Supporting Information).^[^ ^19^ ^]^ The edged‐area has higher curvatures, surface energy and fewer coordinated atoms, making it a less favorable solute for subsequent deposition for PVP adsorption (Figure 1a (i)).^[^ ^20^ ^]^ This difference results in the edges of the AgNPs being more exposed, and accordingly, Au^3+^ etching‐induced galvanic exchange primarily occurs at this area (Figure 1a (ii), left). Tannic acid, with multiple hydroxyl and phenolic structures, results in the tannic acid‐capped AgNPs exhibiting strong reducing properties. In the case of Ag^+^ accumulation and tannic ligand reduction (Figure 1a (ii), right), nanosatellites were formed at the specific edge regions without any additional reducer introduction. Figure 1b shows TEM images of ES‐AuAgNPs synthesized with varying concentrations of HAuCl_4_ at 5 mm intervals (Figure S2 (i), Supporting Information), followed by an increased number of nanosatellites and an enlargement of the hollow size, along with a red‐shift and broadening of the LSPR peak (Figure S2 (ii), Supporting Information). Figure S3 (Supporting Information) clearly indicates that the nanosatellites preferentially grow on the edge regions, and the ES‐AuAgNPs exhibited a more uniform morphology under 15 mm HAuCl_4_. The optimal PVP molar mass ranges from 24 000 to 40 000 g mol^−1^. Below this threshold, etching predominates, resulting in non‐uniform nanosatellite formation at the edge regions. Conversely, exceeding this range favors deposition, which suppresses the development of satellite‐like morphologies (Figure S4, Supporting Information).

The selective affinity of PVP toward Ag, and the reductive nature of the tannic acid ligand, these two parameters, are crucial for the synthesis of ES‐AuAgNPs. To evidence our hypothesis, molecular dynamics simulations were first employed to investigate the blocking behavior between various surfactants and AgNPs capped by different ligands (Figure S5, Supporting Information). i) Citrate‐capped Ag with PVP and ii) tannic acid‐capped Ag with SDS were used as the controls to compare with iii) our method, tannic acid‐capped Ag with PVP. The simulated results show that PVP has a stronger affinity toward tannic acid‐capped Ag, which can be attributed to PVP's electrically neutral nature and the formation of multiple hydrogen bonds with tannic acid.^[^ ^21^ ^]^ Figure S6a (i) (Supporting Information) presents an SEM image of citrate‐capped AgNPs treated by PVP and HAuCl_4_, only the etching process was excited to form the hollow structure (Figure S6a (ii), Supporting Information), indicating the citric acid ligand cannot in situ reduce the Ag⁺ or Au^3^⁺ to form the satellite structure. We further studied tannic acid capped‐AgNPs reacted with SDS and HAuCl_4_ (Figure S6b (i), Supporting Information). The SDS shows weak binding affinity toward AgNPs and thus possesses poor blocking efficiency, failing to achieve dense nanosatellite wherein the edge area (Figure S6b (ii), Supporting Information).^[^ ^22^ ^]^ Together, the formation of nanosatellites necessitates the participation of PVP blocking and tannic acid ligands. Of course, introducing additional reducing agents could lead to the formation of nanosatellite structures.^[^ ^23^ ^]^ However, numerous parameters need to be optimized for balancing the etching and reduction process, and that of nanosatellites may not preferentially grow in edged‐regions with strong plasmonic effects.

### 2.2 Characterization of ES‐AuAgNPs

**Figure 2:**
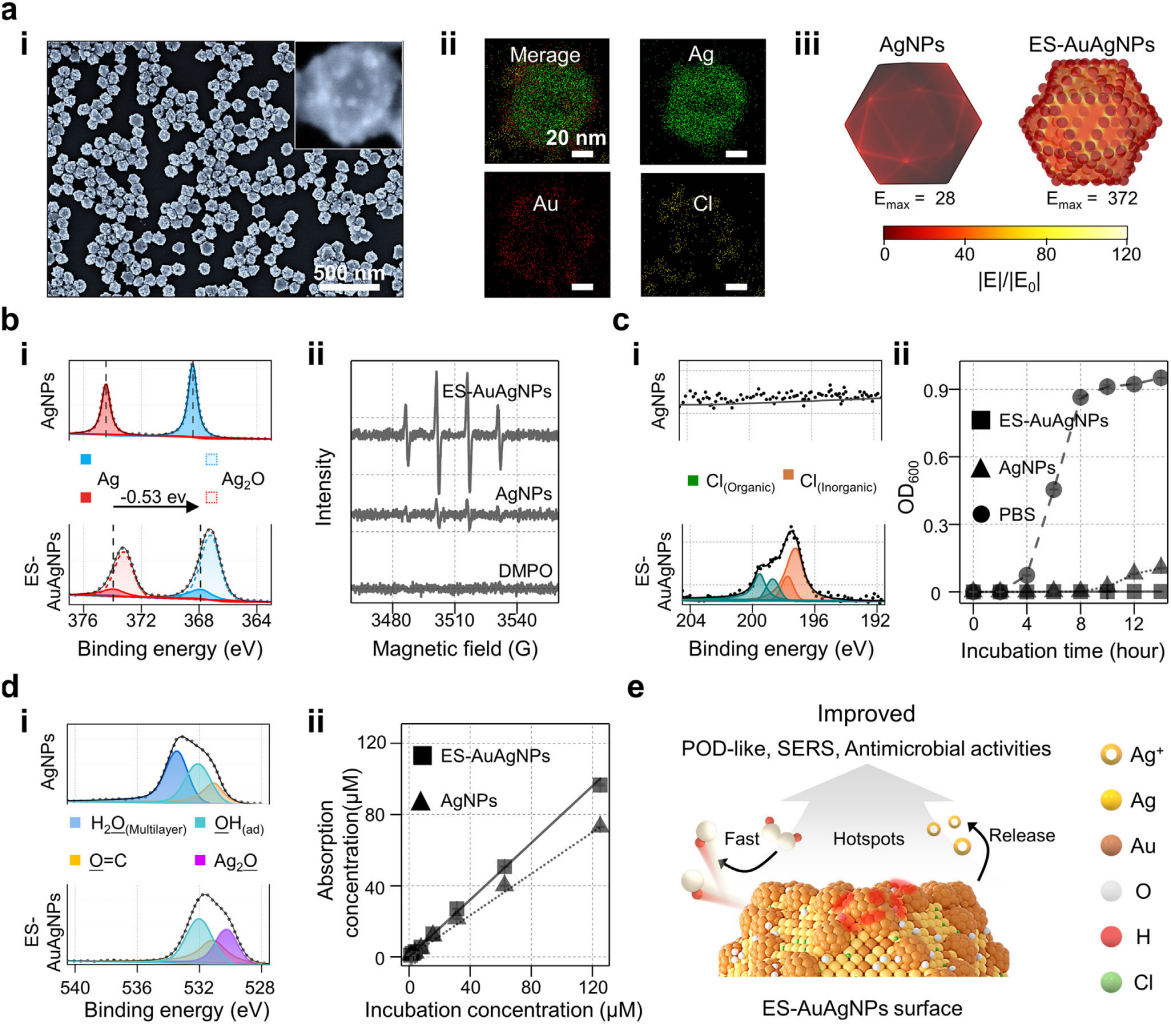
Structure, composition and surface characterization. a) (i) SEM image of ES‐AuAgNPs along with a magnified size, (ii) EDS mapping of the ES‐AuAgNPs, and (iii) electric field simulation for AgNP and ES‐AgAuNP. b) (i) XPS spectra of the Ag 3d spectra and (ii) EPR spectra of •OH radical. c) (i) XPS spectra of the Cl 2p spectra, and (ii) antibacterial ability toward *Vibrio parahaemolyticus*. d) (i) XPS spectra for the O 1s spectra, and (ii) the molecular adsorption kinetics. e) Summary of the properties of ES‐AuAgNPs.

A series of characterization experiments was conducted to investigate whether ES‐AgAuNPs exhibit the desired properties and to elucidate their performance improvement over AgNPs. In the SEM imaging (**Figure** **2**a (i)), the diameter size of ES‐AuAgNPs was determined to be ≈80 nm, and there are 106 nanosatellites in a single particle with a ≈5 nm‐sized gap. We selected 80 nm AgNPs for the synthesis of ES‐AuAgNPs due to their optimal balance of morphology and construction, while smaller AgNPs yield ES‐AuAgNPs with fewer satellites, and larger AgNPs lead to unstable morphologies. According to the EDS mapping results (Figure 2a (ii)), ES‐AuAgNPs were found to contain both Au and Cl elements, distinguishing them from their template, and the Ag‐to‐Au ratio was determined to be 7:3 by ICP‐OES. We found that the Au element is predominantly distributed in the nanosatellite regions according to the HAADF‐EDS mapping (Figure S7 (i), Supporting Information). The presence of Cl is attributed to the deposition of AgCl (Figure S7 (ii), Supporting Information), and the nanoscale deposition of AgCl can improve the surface properties of ES‐AuAgNPs, enabling the sustained release of silver ions.^[^ ^24^ ^]^ Given these dimensions and element compositions, finite‐element‐method (FEM) simulations were carried out to analyze the electric field distribution on the surface of ES‐AuAgNPs. As shown in Figure 2a (iii), the hotspots were formed within the nanogaps between the nanosatellite‐to‐nanosatellite and nanosatellite‐to‐edge. As a result, the E_max_ value of ES‐AgAuNP reached 372, much higher than that of the AgNPs (E_max_ = 28). We have successfully alloyed Ag and Au elements, constructed numerous nanogap structures, and improved the surface properties, all converging into the ES‐AgAuNPs, which can correspondingly enhance their catalytic activity, SERS capabilities, and antibacterial properties.

In addition to these reshaped structuration and changes in elemental composition, we believe that alterations in the surface elemental chemical/electronic states can further improve these properties.^[^ ^25^ ^]^ Then, we employed XPS technology to analyze the elemental chemical/electronic state of ES‐AuAgNPs. The Ag 3d XPS spectra exhibit that the ES‐AuAgNPs presented two distinct characteristics toward AgNPs (Figure 2b (i)), the Ag_2_O deposition (530.2 eV) and a negative binding energy shift (−0.53 eV) in the Ag 3d_5/2_ and Ag 3d_3/2_ peaks.^[^ ^26^, ^27^ ^]^ The oxidation and de‐energization of Ag enhance the electron transfer capabilities, which in turn further boost the POD‐like catalytic activity of ES‐AuAgNPs.^[^ ^28^ ^]^ Next, we employed electron paramagnetic resonance (EPR) technology to verify this improvement (Figure 2b (ii)). Note that a strong EPR signal wherein ES‐AuAgNPs, referring to a 1:2:2:1 signal corresponding to the DMPO–•OH, was detected.^[^ ^29^ ^]^ Their significant POD‐like activity leads to the rapid H_2_O_2_ decomposition and the generation of a large number of •OH radicals. To prove the role of Ag_2_O deposition in catalytic activity, ES‐AuAgNPs were treated with NH_3_‐H_2_O to release Ag_2_O selectively. As evidenced by EPR spectroscopy (Figure S8, Supporting Information), this removal correlates with a significant decrease in the POD‐like catalytic activity. In the Cl 2p XPS spectra, bactericidal organic chlorine deposition was observed on the surface of ES‐AuAgNPs (Figure 2c (i)). The incorporation of such antibacterial components dramatically improves their antibacterial property over AgNPs (Figure 2c (ii)).^[^ ^30^ ^]^ Figure 2d (i) shows the O 1s state, we found the H_2_O_(Multilayer)_ absence in the ES‐AuAgNPs' surface, whereas AgNPs retain it.^[^ ^28^ ^]^ The removal of these water layers effectively eliminates the barrier to surface interactions, and thus significantly enhances surface accessibility.^[^ ^31^ ^]^ We further conducted molecular adsorption experiments to evaluate its molecular adsorption performance. Figure 2d (ii) demonstrates the molecular adsorption kinetics for both types of NPs, the well‐accessible surface of ES‐AuAgNPs allows for a more efficient interaction with the adsorbate, and thus leads to increased adsorption of molecules onto their surface. These changes, either in reshaped structuration or updated surface elemental composition/state (Figure 2e), synergistically enhance the overall POD‐like catalytic activity, SERS capabilities, and antibacterial properties of ES‐AuAgNPs.

### 2.3 Performance Evaluation of ES‐AuAgNPs

**Figure 3:**
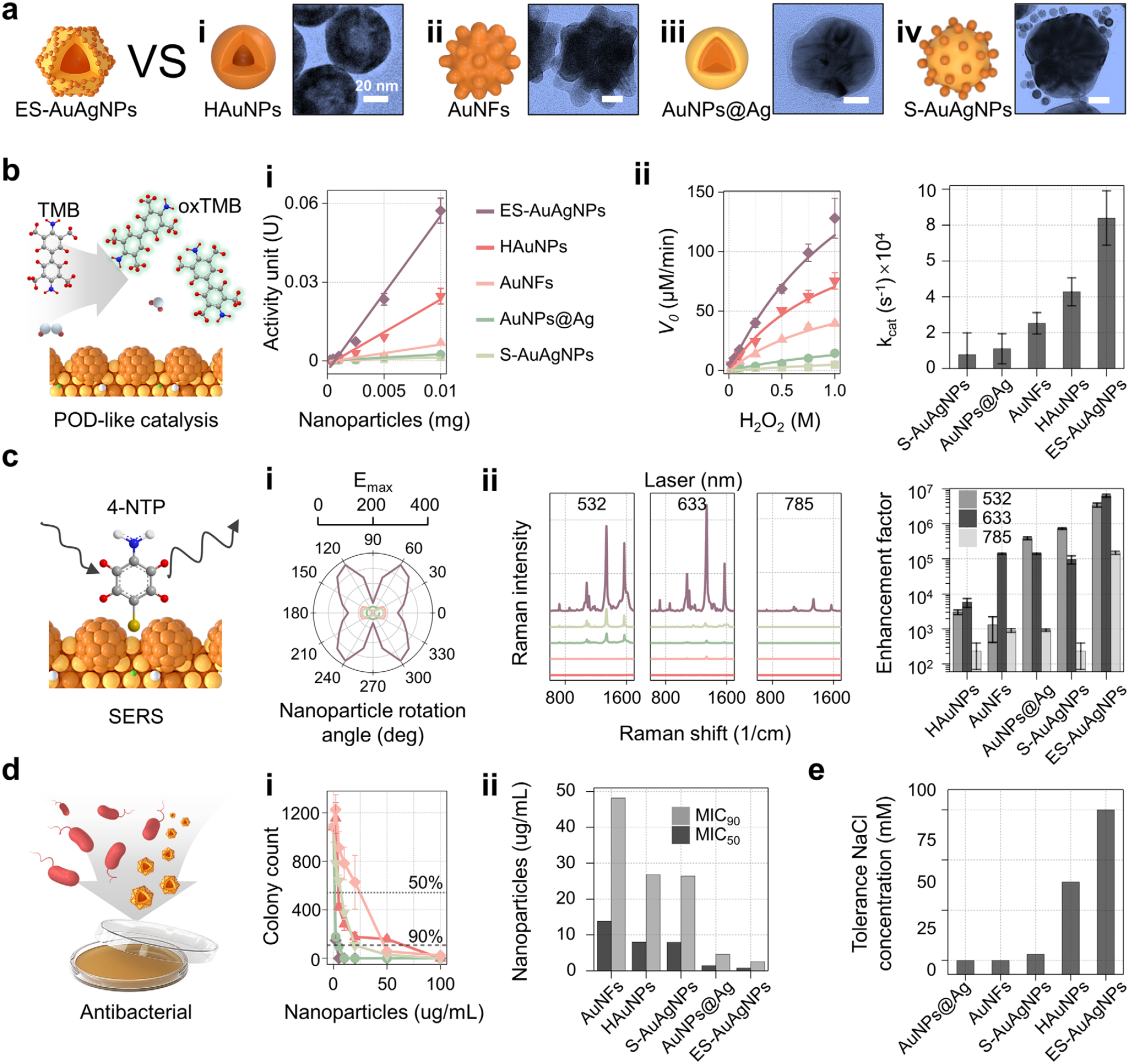
Performance evaluation of ES‐AuAgNPs toward a) (i) HAuNPs, (ii) AuNFs, (iii) AuNPs@Ag, and (iv) S‐AuAgNPs. b) POD‐like activity evaluation: the catalytic (i) activity units versus weight of NPs, (ii) kinetics and constants (k_cat_) for each nanoparticle. c) SERS activity evaluation: (i) the simulated |E_max_| value of each nanoparticle under different spin orientations; (ii) the Raman spectra and the enhancement factor (EF) of each nanoparticle under different laser wavelengths. d) Antibacterial activity evaluation: *Vibrio parahaemolyticus* were incubated with each nanoparticle, (i) colony number and (ii) MIC_50_/MIC_90_ value under varied NPs dosages. e) Stability evaluation by determining their NaCl tolerance concentration.

The multifunctional properties of ES‐AuAgNPs were systematically studied and then compared with four typical plasmonic NPs (**Figure** **3**a), including hollow AuNPs (HAuNPs), Au nanoflowers (AuNFs), satellited‐AuAgNPs (S‐AuAgNPs) and silver‐shelled AuNPs (AgNPs@Ag). Figure S9 (Supporting Information) shows the characteristics of these four NPs, they are similar to ES‐AuAgNPs, but differ only in alloy composition, nanogap structure, or surface accessibility (Table S1, Supporting Information). The motivation behind utilizing them to perform these comparisons is to investigate whether the combination of these various features in ES‐AuAgNPs results in a synergistic enhancement of its multi‐performances.

Their POD‐like catalytic activities were evaluated based on the colorimetric reactions of NPs that oxidize colorless TMB to blue–green‐oxTMB (Figure 3b). We first calculated the POD‐like specific activity (SA, U/mg) according to the nanozyme evaluation criteria proposed by Yan's group.^[^ ^32^ ^]^ Figure S10 (Supporting Information) records time‐differenced colorimetric reactions under varying NP concentrations, which were utilized to build a calibration curve for the SA calculation (Figure 3b (i)). ES‐AuAgNPs exhibited the highest SA at 5.91328 U/mg. Alloyed cobalt element gave HAuNPs satisfactory catalytic activity.^[^ ^33^ ^]^ S‐AuAgNPs, structurally most similar to our NPs but with worse surface accessibility due to additional PEI and CTAC ligands, showed the lowest catalytic efficiency.^[^ ^34^ ^]^ Figure 3b (ii) shows the catalytic kinetics of five NPs at varying H_2_O_2_ concentrations (Figure S11, Supporting Information), along with their calculated catalytic rate constants (kcat). Still, ES‐AuAgNPs demonstrated an exceptional kcat value of 83947.809 s^−1^, much higher than that of commercial horseradish peroxidase (HRP, ≈23400 s^−1^).^[^ ^35^ ^]^ We also evaluated the overall catalytic performance using the reaction of 4‐nitrophenol (4‐NP) can be reduced to 4‐aminophenol (4‐AP) in the presence of NPs and NaBH_4_. Among the five tested NPs, the apparent rate constants (K_app_) were measured, with 1.3 × 10^−2^ s^−1^ (ES‐AuAgNPs), 4.2 × 10^−3^ s^−1^ (HAuNPs), 2.9 × 10^−3^ s^−1^ (AuNFs), 1.5 × 10^−3^ s^−1^ (AuNPs@Ag), and 1.22 × 10^−3^ s^−1^ (S‐AuAgNPs).

The SERS capability was evaluated by electric field simulation and Raman intensity measurement (Figure 3c). The specific wavelength for plasmonic resonance excitation and the heterogeneous distribution of hot spots may influence corresponding NPs' SERS performance.^[^ ^36^ ^]^ As shown in Figure 3c (i), the generation of hotspots over the entire surface and the broadening of LSPR lead to the ES‐AuAgNPs' E_max_ value significantly exceeding that of other NPs from any angle. Figure 3c (ii) presents the Raman intensity and corresponding SERS enhancement factors (EF) of the five NPs after 4‐NTP conjugation. ES‐AuAgNPs exhibit exceptional EF across various laser wavelengths, indicating their non‐dependence on specific wavelengths and their potential for single‐molecule detection, with a maximum EF approaching 10^7^.^[^ ^37^, ^38^ ^]^ In contrast, HAuNPs, despite high POD‐like activity, demonstrated the poorest SERS performance given by the limited hotspot generation (Figure S12, Supporting Information).

We further incubated five NPs with *Vibrio parahaemolyticus* to evaluate their antibacterial performance (Figure S13, Supporting Information). Figure 3d (i) shows that ES‐AuAgNPs have the most effective antibacterial performance, requiring minimal dosages to inhibit 50% (MIC_50_ = 0.77077 µg mL^−1^) or 90% (MIC_90_ = 2.55255 µg mL^−1^) of bacteria (Figure 3d (ii)).^[^ ^39^ ^]^ These values are close to those of the commercial antibiotic (gentamicin, MIC_50_ = 0.5 and MIC_50_ = 2 µg mL^−1^).^[^ ^40^ ^]^ In contrast, the AuNS, composed of stable mono‐component Au, exhibited the poorest bactericidal activity. The stability of nanoparticles (NPs) critically impacts their application potential, particularly for on‐site detection in seawater samples. Therefore, we proceeded to evaluate the salt tolerance of ES‐AuAgNPs, along with that of four other types of NPs. The residual tannic acid capping, AgCl deposition, and strong negative electrostatic potential endow ES‐AuAgNPs with considerable stability (Figure S9 (iv), Supporting Information), resulting in a remarkable tolerance to NaCl concentrations (Figures 3e; S14, Supporting Information).^[^ ^41^ ^]^

These systematic comparisons demonstrate that the Ag/Au alloy composition, numerous nanogap structures, and well‐surface accessibility can synergistically enhance the POD‐like catalytic, SERS, and antimicrobial activities, as well as the stability of ES‐AuAgNPs. These findings indicate that ES‐AuAgNPs are well‐balanced multifunctional NPs, suitable for building versatile platforms.

### 2.4 Principle of ES‐AuAgNPs‐Based Versatile Platform

*Vibrio* diseases are the leading culprits behind mass mortality in marine aquaculture animals (especially the white shrimp), characterized by rapid onset (< 12 h), severe progression (> 90% mortality rate), and are caused by multiple *Vibrio* species acting in concert.^[^ ^42^ ^]^ Among these, *Vibrio parahaemolyticus*, *Vibrio vulnificus*, and *Vibrio alginolyticus* are major representatives.^[^ ^43^ ^]^ Beyond their aggressive pathogenicity, these bacteria share similar 16S rDNA genes yet often manifest distinct symptoms. Consequently, corresponding detection methods must be both rapid and sensitive, with the additional capability to differentiate between bacterial strains. Real‐time qPCR is the gold standard for *Vibrio* detection, whereas its lengthy procedure (> 4 hours) hinders the timely prevention of infections.^[^ ^44^ ^]^ Though some high sensitivity on‐site detection technologies have been developed, they often neglect the post‐processing of samples associated with efficient sterilization. To counteract the threats posed by *Vibrio* species and avoid secondary infections, we constructed an ES‐AuAgNPs‐based versatile platform capable of simultaneously screening, validating, and eliminating the three aforementioned *Vibrio* strains with minimal resource consumption.

**Figure 4:**
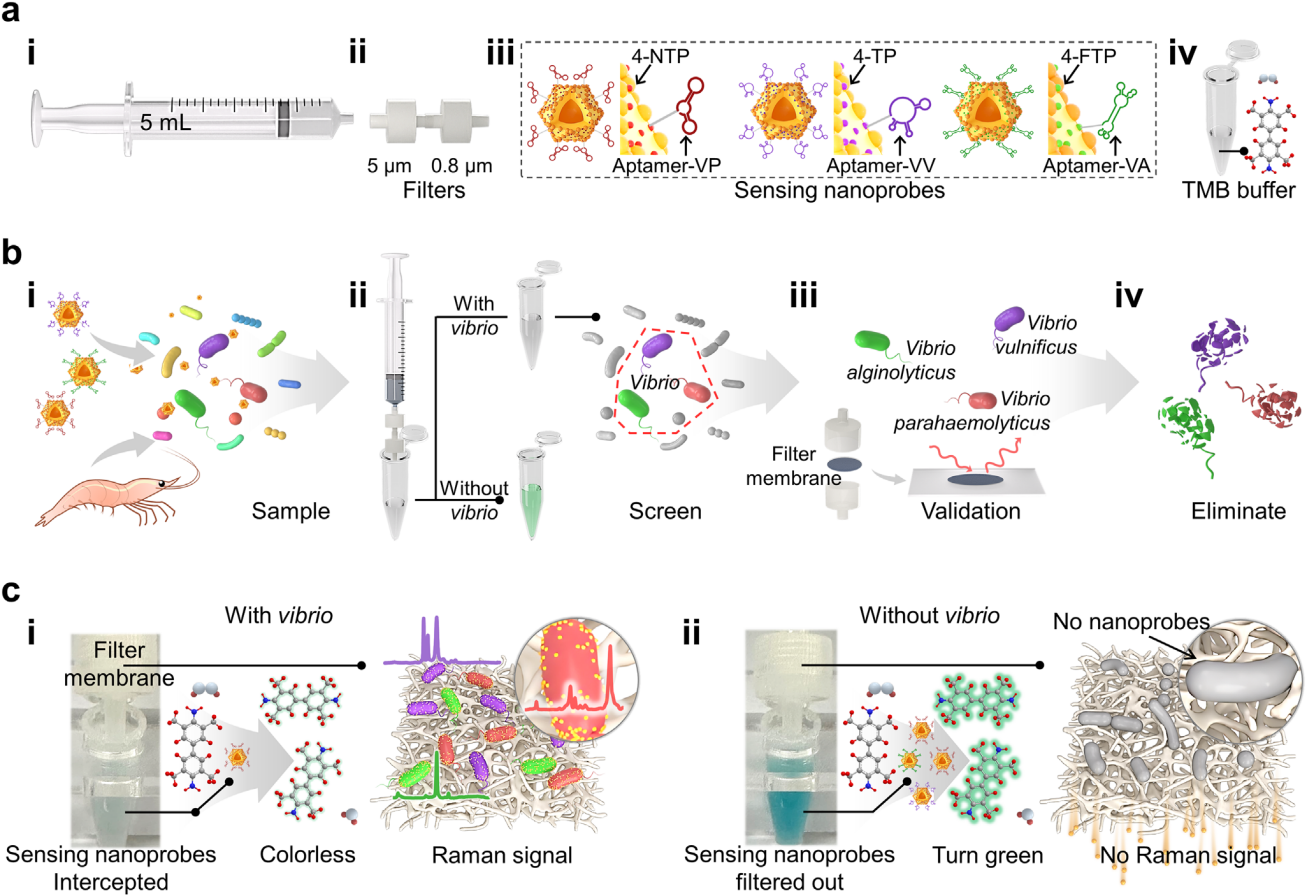
a) Components: (i) a syringe, (ii) two filters, (iii) sensing nanoprobes, (iv) TMB buffer. The sensing nanoprobes fabricated by ES‐AuAgNPs conjugated with different Raman reporters and aptamers, enable them to specifically bind to *Vibrio parahaemolyticus*, *Vibrio vulnificus*, and *Vibrio alginolyticus*, respectively. b) Assay scenario: (i) Samples were extracted and reacted with sensing nanoprobes, (ii) *Vibrio* presence was screened by colorimetric detection, and (iii) *Vibrio* types were classified by SERS‐based assay. (iv) Sterilization was achieved through the antibacterial properties of the sensing nanoprobes. c) Principle: (i) with and (ii) without *Vibrio* bacteria.

**Figures** **4**a and S15a (Supporting Information) show the components of the constructed platform by ES‐AuAgNPs. It incorporates a i) syringe, ii) 5 µm/0.8 µm pore‐sized filters, iii) sensing nanoprobes, and iv) a collection tube filled with TMB buffer. Wherein, the syringe aims to collect the reaction solution and transfer the reacted sample to a collection tube. The 5 µm pore‐sized filter removes large particulate impurities (Figure S15b (i), Supporting Information), the 0.8 µm pore‐sized filter intercepts *Vibrio* bacteria and then filters out unreacted nanoprobes into the collection tube (Figure S15b (ii), Supporting Information). The sensing nanoprobes comprise ES‐AuAgNPs conjugated with a Raman reporter and a DNA aptamer, allowing for specific binding to three types of *Vibrio* species (Table S2, Supporting Information).^[^ ^45^, ^46^, ^47^ ^]^ This platform simultaneously enables (i) sample preparation; (ii) on‐site screening of presented *Vibrio parahaemolyticus*, *Vibrio vulnificus*, and *Vibrio alginolyticus*; (iii) classification of *Vibrio* strains; and (iv) in situ sterilization to eliminate residual bacteria post‐detection (Figure 4b).

Figure 4c illustrates the screening and classification mechanism in our proposed platform. (i) In the presence of *Vibrio* bacteria, sensing nanoprobes‐bacteria complexes are formed by the specific “aptamer‐bacterial surface protein” interaction (Figure S16, Supporting Information). Most of the sensing nanoprobes form complexes that are accumulated on the 0.8 µm filter membrane (Figure S17, Supporting Information), hence, a tiny portion of unreacted sensor nanoprobes flows into the TMB‐rich collection tube, insufficiently converting TMB to oxTMB, resulting in a nearly colorless solution in the collection tube. The filter membrane loaded with sensing nanoprobes‐bacteria complexes was transferred for the SERS‐based assay. Each sensing nanoprobe was modified with a different Raman reporter, exhibiting a distinct Raman fingerprint spectrum. Therefore, *Vibrio* strains can be identified and classified based on the detected Raman characteristic peaks (Figure S18, Supporting Information). Among them, the Raman peaks of 4‐NTP (1341 cm^−1^), 4‐TP (998 cm^−1^), and 4‐FTP (813 cm^−1^) correspond to *Vibrio parahaemolyticus*, *Vibrio vulnificus*, and *Vibrio alginolyticus*, respectively. In the absence of Vibrio bacteria (Figure 4c (ii)), the specific sensing nanoprobes do not interact with other bacteria, leading to a large accumulation in the collection tube. As a result, this causes noticeable colorimetric changes and weaker Raman signals. Therefore, the quantitative relationship between absorbance, *Vibrio* concentration, and Raman intensity can be utilized for the precise analysis of *Vibrio* concentration and type.

### 2.5 Performance of ES‐AuAgNPs‐Based Versatile Biosensor

**Figure 5:**
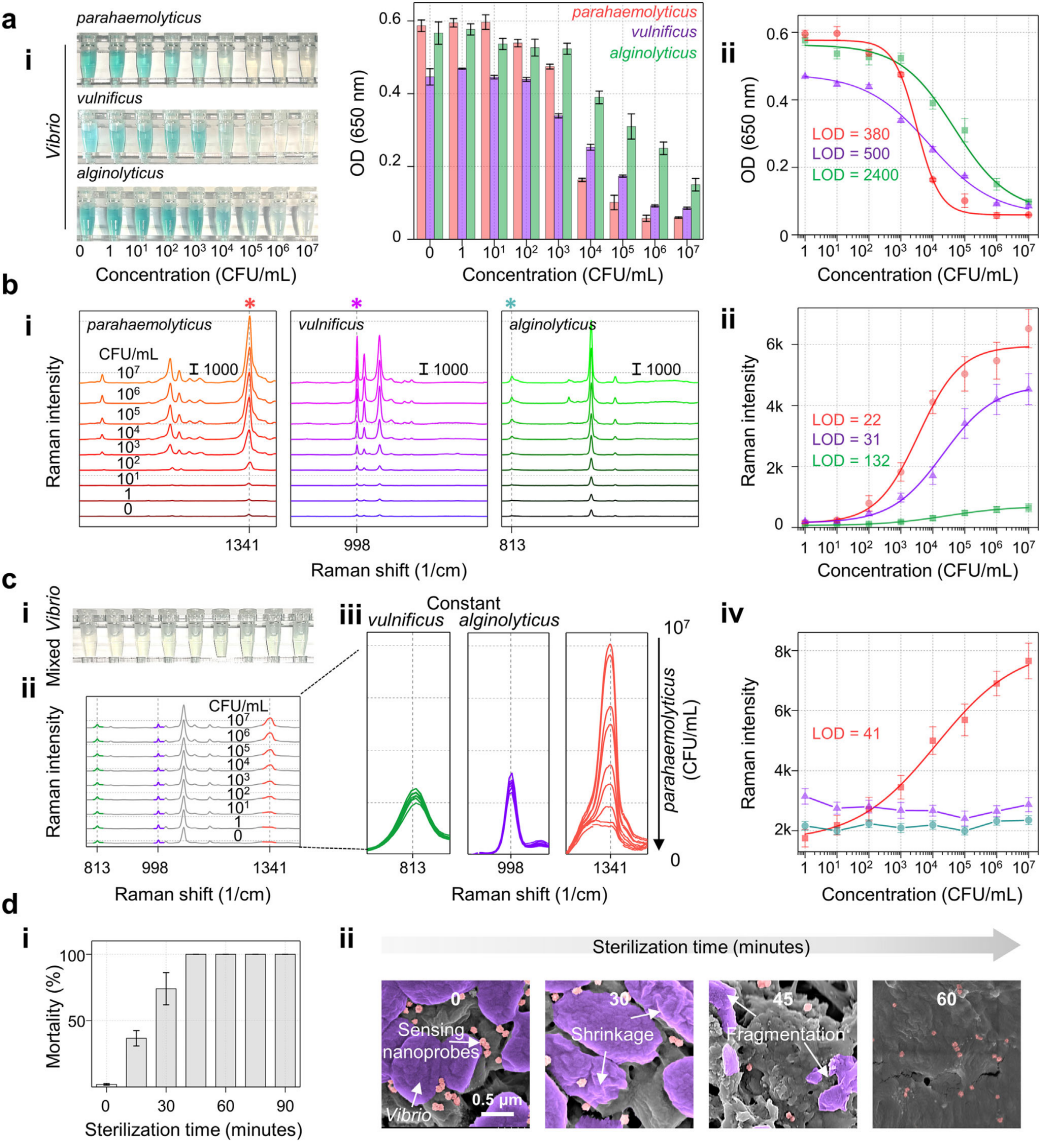
Performance of ES‐AuAgNPs‐based biosensor. a) Screen: (i) photography, (ii) OD determination, and (iii) calibration curves at various *Vibrio* bacterium concentrations. b) Validation: (i) Raman spectra and (ii) calibration curve. The scale bar presents the Raman intensity. c) Sensing ability in mixed *Vibrio* condition: varied amounts of *Vibrio parahaemolyticus* were spiked into the other two *Vibrio* bacteria (10^7^ CFU per mL) mixture. (i) the screening photography, (ii) the identification involves Raman spectra with its (iii) characteristic Raman peak, and the (iv) calibration curve. d) Bacteria elimination, (i) time‐difference of mortality for *Vibrio parahaemolyticus* exposed to sensing nanoprobes, and (ii) corresponding SEM imaging of the filter membrane.

The sensitivities of screening and validating were first evaluated. Different concentrations of *Vibrio parahaemolyticus*, *Vibrio vulnificus*, and *Vibrio alginolyticus* were introduced separately into our developed platform. In the screening detection, the sensing nanoprobes in the collection tube gradually decreased as the *Vibrio* concentration increased, causing the color to shift from blue–green to colorless (**Figure** **5**a (i)). Figure 5a (ii) shows the calibration curves between detected OD value and *Vibrio* concentrations, and the calculated limit of detection (LOD) was 350 CFU per mL for *Vibrio parahaemolyticus*, 500 CFU per mL for *Vibrio vulnificus*, and 2400 CFU per mL for *Vibrio alginolyticus*.^[^ ^48^ ^]^ These LODs from screening were significantly lower than the concentrations (10^4^ CFU per mL) required for *Vibrio* to exhibit their toxicity, demonstrating the platform's capability for rapid warning of *Vibrio* spreading.^[^ ^49^ ^]^

In the classifying identification, Raman mapping was performed to measure each membrane's Raman signal (Figure S19, Supporting Information). Figure 5b (i) shows the averaged Raman spectra from Raman mapping. Their signal intensity is proportional to the *Vibrio* concentration, given the increasing number of sensing nanoprobe‐*Vibrio* complexes retained on the filter membrane. Figure 5b (ii) presents the built calibration curves, based on which the determined LODs of SERS‐based validation for *Vibrio parahaemolyticus*, *Vibrio vulnificus*, and *Vibrio alginolyticus* are 22 CFU per mL, 31 CFU per mL, and 132 CFU per mL, respectively. This high sensitivity allows this platform to precisely distinguish different *Vibrio* strains and early diagnosis of *Vibrio* infections.^[^ ^50^ ^]^

To test the reproducibility associated with the cross‐reactivity in our platform, we evaluated the detection sensitivity of the platform by mixing varying concentrations of *Vibrio parahaemolyticus* with 10^7^ CFU per mL of *Vibrio vulnificus* and *Vibrio alginolyticus*. In the presence of the other two *Vibrio* species, all the collection tubes show colorlessness (Figure 5c (i)). Figure 5c (ii) presents the detected Raman spectra, merging the characteristic peaks of the three *Vibrio* strains. The Raman characteristic peak intensities belonging to *Vibrio vulnificus* and *Vibrio alginolyticus* remain constant, while the peak intensity attributed to *Vibrio parahaemolyticus* increases with its concentration (Figure 5c (iii)). Under this condition, the LOD for *Vibrio parahaemolyticus* was estimated to be 41 CFU per mL, closely approximating the ideal LOD of 22 CFU per mL (Figure 5c (iv)). Similarly, the platform's sensitivity remains consistent when varying concentrations of *Vibrio vulnificus* (Figure S20a, Supporting Information) or *Vibrio alginolyticus* (Figure S20b, Supporting Information) are mixed with other *Vibrio* bacteria. These data confirm that our proposed platform exhibited high reproducibility without severe cross‐reactivity.

Meanwhile, four environmental bacteria commonly used in aquaculture and two marine *Vibrio* bacteria with similar 16S rDNA sequences to our target were employed to evaluate the selectivity of our platform.^[^ ^51^ ^]^ The screening detection covers all three target bacteria and shows no response to non‐target bacteria (Figure S21 (i), Supporting Information). The Raman classification exhibits higher selectivity, identifying individual *Vibrio* bacteria from a variety of bacteria (Figure S21 (ii), Supporting Information). This dual‐detection mode enables our platform to rapidly screen three common *Vibrio* bacteria in complex microbial environments and further determine their type.

Lastly, we assessed the bactericidal efficiency by determining the sterilization time when the *Vibrio parahaemolyticus* was treated by the sensing nanoprobes. When the exposure time between *Vibrio* and sensing nanoprobes reaches 45 min (Figure 5d (i)), no *Vibrio* survives, thus confirming a sterilization time of 45 min. The corresponding time‐differenced SEM images (Figure 5d (ii)) illustrate that *Vibrio* biofilm gradually shrinks, starting at 30 min. By 45 min, the bacteria lose their typical morphological characteristics and eventually rupture (Figure S22, Supporting Information). This is comparable to conventional UV sterilization techniques (30 min) but with lower resource consumption. Although the sterilization time is longer compared to hypochlorite‐based disinfectants, the sensing nanoprobes fabricated with ES‐AgAuNPs exhibit remarkable durability and stability.^[^ ^52^ ^]^ Importantly, the ES‐AuAgNPs‐based portable device combines additional rapid screening and multiple typing capabilities, significantly enhancing overall efficiency and offering a much faster process than conventional detection methods.

The workflow of our proposed ES‐AuAgNPs‐based versatile platform is summarized as follows: Samples are collected from the intestines of aquaculture animals, such as shrimp, and mixed with sensing nanoprobes. The mixture is drawn into a syringe and then equipped with dual filters for filtration. The filtered liquid is collected into a tube containing TMB for colorimetric detection. After 45 min, the sample can either be discarded or subjected to further SERS analysis to identify the *Vibrio* species. In practical application, once the *Vibrio* species are detected by the screening assay, immediate control measures are implemented, such as disinfecting aquaculture ponds, or using antibiotics. Subsequent SERS validation then confirms the identity of the *Vibrio* strain for targeted sterilization, preventing its re‐emergence. Furthermore, the additional post‐sample processing can be removed due to the sensor's in situ sterilization capability.

## 3 Conclusion

In summary, we have synthesized a unique multifunctional nanomaterial, that are ES‐AuAgNPs, which exhibit robust capabilities in POD‐like catalytic, SERS, antibacterial activity, and stability. Based on this, we developed a versatile platform integrating screening, classification, and elimination of three prevalent marine *Vibrio* bacteria. This platform exhibits superior analytical performance in terms of sensitivity, reproducibility, and selectivity. From initial sample extraction to sample sterilization, the entire process takes ≈1 h, much lower than that of conventional methods (over 5 h). The strategy and methodology for synthesizing ES‐AgNPs, along with the construction of this versatile platform, pave the way for public health care and environmental monitoring against the pandemic of pathogenic bacteria.

## 4 Experimental Section

### Materials and Instruments

The specific materials and instruments used in this work are comprehensively detailed in the Supporting Information. All chemicals used were of analytical grade, and Milli‐Q water (18.2 MΩ) was employed for all experiments.

### Preparation of Tannic Acid‐Capped AgNPs

Eighty‐nanometer tannic acid‐coated AgNPs were used as sacrificial templates, and their synthesis followed a previously published method with minor modifications.^[^ ^53^ ^]^ 0.14705 g of SC and 0.17012 g of tannic acid were dissolved in 100 mL of Milli‐Q water and heated to 90 °C under vigorous stirring (600 rpm). 1 mL of 25 mm AgNO_3_ was added to the mixture, and the reaction was maintained at 90 °C for another 30 min. The reaction mixture was diluted by extracting 50 mL of the sample and adding 40 mL of water. Then, 500 µL of SC (25 mm), 1.5 mL of tannic acid (2.5 mm), and 1 mL of AgNO_3_ (25 mm) were sequentially added at 1‐minute intervals. This addition sequence was repeated for six cycles. By repeating these processes, dilution (extracting 50 mL of the mixture and adding 48 mL of water) and addition (six cycles of SC, tannic acid, and AgNO_3_), up to twice, ≈80 nm diameter‐sized AgNPs with tannic acid‐capped were finally synthesized. The as‐synthesized AgNPs were centrifuged and washed twice (4000 rpm, 10 min). After redispersing in water, the volume of AgNPs was adjusted to 100 mL for subsequent experiments.

### Preparation of ES‐AuAgNPs

One milliliter of 0.2 nm tannic acid‐capped AgNPs (approximately OD = 1) was sequentially mixed with 3 µL of 10% PVP and 1 µL of 15 mm HAuCl_4_. After vigorous shaking for 30 s using a vortex mixer, the mixture was kept at ambient conditions for 30 minutes to form ES‐AuAgNPs. Finally, these NPs were washed twice with Milli‐Q water to remove residual reagents.

### ChatGPT for Grammar Verification and Translation

In preparing this manuscript, the authors used an AI‐based language model, ChatGPT, developed by OpenAI, for grammar polishing.

## Conflict of Interest

The authors declare no competing financial interest.

## Author Contributions

Y.W. designed, performed and analyzed the main experiments. J.C. performed the experiments associated with conventional NPs synthetization. L. B. analyzed antibacterial evaluation results. Z. Z., X. W. and L. F. analyzed the *Vibrio* detection results. Q.Y. funding acquisition, conceptualization, and written original draft. J.C. and L. C. funding acquisition, draft– review & editing, and supervision. All authors participated in the manuscript discussions and approved the final version.

## Supporting information



Supporting Information

## Data Availability

The data that support the findings of this study are available from the corresponding author upon reasonable request.;
