# Supplementary material for: Multifunctional Edged‐Satellite AuAg Nanoparticles‐Based Integration Platform for Screening, Validation, and Elimination of Vibrio Bacteria
Source: Adv Sci (Weinh). 2025 Nov 5;13(3):e16240. doi: 10.1002/advs.202516240 (PMC12806398; doi:10.1002/advs.202516240)
Supplement: Supplementary file 1 — Supporting Information [file ADVS-13-e16240-s001.docx]

Supporting Information

Multifunctional Edged-satellite AuAg Nanoparticles-based Integration Platform for Screening, Validation, and Elimination of *Vibrio* Bacteria

Yixuan Wu^1,2^, Jiadong Chen^2^, Liyan Bi^3^, Zhiyang Zhang^1,2^, Xiaoyan Wang^3^, Longwen Fu^1^, Qian Yang^4^*, Jaebum Choo^2^*, and Lingxin Chen^1,3,4^*

*^1^ Key Laboratory of Coastal Environmental Processes and Ecological Remediation, Yantai Institute of Coastal Zone Research, Chinese Academy of Sciences, Yantai 264003, China*

*^2^ Department of Chemistry, Chung-Ang University, Seoul 06974, South Korea*

*^3^ School of Special Education, School of Pharmacy, Binzhou Medical University, Yantai, 264003, China*

*^4^ The Institute for Advanced Study, Shaoxing University, Shaoxing, 312000, China*

Y. Wu, Z. Zhang, L. Fu, L. Chen

Key Laboratory of Coastal Environmental Processes and Ecological Remediation, Yantai Institute of Coastal Zone Research, Chinese Academy of Sciences, Yantai, 264003, China

E-mail: lxchen@yic.ac.cn

Y. Wu, J. Chen, J. Choo

Department of Chemistry, Chung-Ang University, Seoul, 06974, South Korea

E-mail: jbchoo@cau.ac.kr

L. Bi, X. Wang, L. Chen

School of Special Education, School of Pharmacy, Binzhou Medical University, Yantai, 264003, China

1. Yang, L. Chen

The Institute for Advanced Study, Shaoxing University, Shaoxing, 312000, China

1. mail: yangqianncu@sina.com

**Contents**

**Experimental section**

Materials and instruments3

Synthesis of plasmonic NPs 5

Simulation 5

Molecular adsorptions6

Performance evaluations 7

Application of ES-AuAgNPs8

References9

**Tables and Figures**

Table S111

Table S212

Figure S113

Figure S214

Figure S315

Figure S416

Figure S517

Figure S618

Figure S719

Figure S820

Figure S921

Figure S1022

Figure S1123

Figure S1224

Figure S1325

Figure S1426

Figure S1527

Figure S1628

Figure S1729

Figure S1830

Figure S1931

Figure S2032

Figure S2133

Figure S2234

**Experimental section**

**1 Materials and instruments**

**1.1 Materials**: Silver nitrate (AgNO_3_), tannic acid (C_76_H_52_O_46_), trisodium citrate (SC), gold chloride trihydrate (HAuCl_4_), polyvinylpyrrolidone (PVP, Mw=24000), sodium dodecyl sulfate (SDS), cobalt chloride (CoCl_2_·6H_2_O), sodium borohydride (NaBH_4_), hydroquinone (C_6_H_6_O_2_), L-ascorbic acid (C₆H₈O₆), cetyltrimethylammonium bromide (CTAB), cetyltrimethylammonium chloride (CTAC), polyethyleneimine (PEI), 3,3',5,5'-tetramethylbenzidine (TMB), hydrogen peroxide (H_2_O_2_, 30%), 4-nitrothiophenol (4-NTP), 4-fluorothiophenol (4-FTP), 4-thiophenol (4-TP), tris (2-carboxyethyl) phosphine hydrochloride (TCEP), and NaCl. Tris-EDTA buffer (TE, pH 7.0, 100×) and PBS buffer (pH 7.4, 100×) were purchased from Sigma-Aldrich (St. Louis, MO, USA). Tetrabutylammonium bis(3,6-dichloro-1,2-benzene-dithiolato) nickelate (TBA-bis-Ni) was purchased from TCI chemicals (Tokyo, Japan). NaAc-HAc buffer (pH=3.6) was prepared from 0.2 M HAc and 0.2 M NaAc·3H_2_O. Milli-Q water (18.2 MΩ cm) was obtained by laboratory equipment from Thermo Fisher Scientific (Waltham, MA, USA). Citrate-capped AgNPs (~80 nm diameter) were purchased from Shenzhen Zhong Ke Nano Technology (Shenzhen, China). 2216E liquid medium and *Vibrio* chromogenic solid medium were purchased from Hopebio Company (Shandong, China). The disposable syringe was purchased from Becton Dickinson Company (Huesca, Spain). 5/0.8 pore-sized membranes and filter heads were purchased from Jinteng Company (Tianjin, China). *Vibrio parahaemolyticus* (BNCC333072), *Vibrio vulnificus* (BNCC337298), *Vibrio alginolyticus* (BNCC337013) and *Vibrio harveyi* (BNCC336937) were purchased from Bena Culture Collection (Henan, China). *Vibrio neocaledonicus*, *Acinetobacter calcoaceticus*, *Bacillus subtilis*, *Bacillus licheniformis*, and *Lactobacillus plantarum* were isolated from the Guangdang River in Yantai and identified through 16S rDNA sequencing.

**1.2 Instruments:** Transmission electron microscopy (TEM) and energy-dispersive X-ray spectroscopy (EDS) element mapping images were captured by a JEM-1400 (JEOL Ltd, Japan) operating at 100 kV. Scanning electron microscope (SEM) images were taken using an S-4800 field emission scanning electron microscope (Hitachi, Japan). Size and quantity measurements were performed using ImageJ software (NIH, Maryland, USA). X-ray diffraction (XRD) measurements were carried out by Bruker D8 Advance (Karlsruhe, Germany). ICP-OES analysis was performed to determine the element content by AGILENT 730 (Santa Clara, CA, USA). X-ray photoelectron spectroscopy (XPS) measurements were carried out using ESCALAB 250xi (Waltham, MA, USA). Electron paramagnetic resonance (EPR) spectroscopy was obtained from Bruker A300 (Bruker, MA, Germany). Ultraviolet−visible (UV−vis) spectra were recorded by a NanoDrop2000/2000C spectrophotometer (Thermo Fisher Scientific, Waltham, MA, USA). Raman measurements were carried out using an inVia Renishaw Raman microscope system (Renishaw, New Mills, UK).

**2 Synthesis of plasmonic NPs**

**2.1 Synthesis of HAuNPs:** The HAuNPs synthetization follows the principle of HAuCl_4_-induced galvanic exchange with cobalt NPs.^1^ First, 1 mL of 0.1 M Na_3_-citrate was added to 100 mL of deionized water and stirred at 500 rpm under an N_2_ atmosphere for 30 minutes. Following this, 140 μL of freshly prepared NaBH_4_ (1.0 M) and 100 μL of CoCl_2_ (0.4 M) were sequentially added to the mixture at 10-second intervals. After an additional 45-minute reaction, 400 μL of HAuCl_4_ solution (0.1 M) was added in 8 aliquots of 50 μL with 2-minute intervals for the HAuNPs formation.

**2.2 Synthesis of AuNFs:** The AuNFs synthetization follows the principle of the heterogeneous reducing ability of hydroquinone.^2^ First, 30 nm sized AuNPs were synthesized as seeds,^3^ and then 2 mL of 0.2 nM AuNPs was dissolved in 40 mL of water. 50 µL of 0.1 M HAuCl_4_ and 100 µL of 0.4 M hydroquinone were added sequentially to the mixture under 550 rpm stirring. After 30 minutes of reaction, the as-prepared AuNFs were washed and kept in ambient conditions.

**2.3 Synthesis of** **S-AuAgNPs:** The S-AuAgNPs synthetization follows the principle of PEI-induced electrostatic interaction.^4^ 3 µL of 10% PEI was added to 1 mL of 10 nm CTAC-coated AuNPs to enhance their positive surface charge.^5^ 100 µL of positively charged AuNPs were then mixed with 1 mL of tannic acid-coated, negatively charged AgNPs (OD = 1) to form satellite-structured S-AuAgNPs.

**2.4 Synthesis of AuNPs@Ag:** The AuNPs@Ag synthetization involves using AuNPs as the core and Ag as the shell.^6^ 60 µL of 0.1 M L-ascorbic acid was added to 5 mL of 0.2 nM AuNPs with a diameter of 60 nm. Subsequently, 60 µL of 10 mM AgNO_3_ was added to the mixture in five separate aliquots under continuous stirring. The AgNO_3_ was reduced by ascorbic acid to form a silver shell on the AuNPs. After an additional 30 minutes of reaction, the as-prepared AuNPs@Ag were then centrifuged and washed.

**3 Simulation**

**3.1 Molecular dynamics simulation:** The simulation was conducted using density functional theory (DFT) to determine the adsorption behavior of SDS and PVP on the Ag {111} surface with different ligands.^7^ The bulk crystalline cells and atomic positions were optimized using the generalized gradient approximation (GGA) with the Perdew-Burke-Ernzerhof (PBE) exchange-correlation functional. An energy cut-off of 360 eV was used for the plane-wave basis set. The parameters for the convergence criteria of geometry optimization were defined as follows: an energy change tolerance (1.0 x 10^-5^ eV/atom), along with the maximized tolerance values of force (0.05 eV/Å), stress (0.03 GPa), and displacement (0.01 Å).

**3.2 Electric field simulation:** This simulation was performed by the finite element method (FEM) to investigate the local electric field distributions on the surface of plasmonic NPs, and we utilized additional software, SolidWorks, to assist in constructing the 3D model. The 3D model was constructed in SolidWorks software with reference to the experimental parameters, and was then imported into COMSOL Multiphysics for the electric field simulation. The incident light wavelengths were 532, 633, and 785 nm, polarized in the x direction and incident in the z direction. The refractive index of the air at these wavelengths was set as 1, and the physical property of Au and Ag was based on reported papers.^8,9^

**4 Molecular adsorptions**

We selected the TBA-bis-Ni molecules (maximum absorption at 900 nm) to study molecular adsorption as it effectively avoids interference from UV absorption peaks and electrostatic reactions in the ES-AuAgNPs and AgNPs. First, various TBA-bis-Ni compound concentrations were measured by a UV-vis spectrophotometer, aiming to create a standard calibration curve for OD value versus concentration. Then, 1 mL 0.5 nM of ES-AuAgNPs and AgNPs were incubated with the Ni (II) compound ranging from 0 to 125 μM. After a 30-minute reaction and centrifugation, the OD value of the supernatant (OD_supernatant_) was measured. Based on the standard calibration and OD_supernatant_, the absorbed concentration of TBA-bis-Ni on the surface of NPs can be determined.

**5 Performance evaluations**

**5.1 POD-like catalytic activity evaluation:** The POD-like specific activity (SA, U/mg), which represents the number of enzyme units per unit mass. 100 μL of different concentrations of NPs (0.5, 1, 5, 10, 50, 100 μg/mL) was added to 700 μL of Na-AC buffer. 100 μL 10 mg/mL TMB was added to the mixture and kept in dark for 1 minute. 100 μL 10 M H_2_O_2_ was added to the mixture, and then the UV-vis absorbance intensity at 652 nm was measured with a 10-second interval. The SA = V / (ε × l) × (ΔA / Δt) / [M], where V=1 cm^3^, ε = 39,000 M^-1^ cm^-1^, l is the path length of light in the cuvette (cm), ΔA/Δt is the initial rate of change in absorbance at 652 nm/min, and [M] is the weight of the plasmonic NPs (mg).

The catalytic constant (kcat) is the maximum number of substrate molecules converted to product per unit time. 100 μL 100 μg/mL of NPs added to 700 μL of NaAc-HAc buffer. 100 μL of 10 mg/mL of TMB substrate was added to the mixture and kept in the dark for 1 minute. 100 μL of different concentration of H_2_O_2_ (10, 7.5, 5, 2.5, 1, 0.5 mol/L) was added to the mixture and the UV-vis absorbance intensity at 652 nm was measured with a 10-second intervals. The formula is given by kcat = V_max_/[E] = (ΔA / Δt) × (Km + [S])) / ([S] × [E]), where [S] is the concentration of H_2_O_2_, [E] is the molar concentration of plasmonic NPs, and Km is the substrate concentration at which the reaction rate is half of V_max_.

**5.2 SERS activity evaluation:** The SERS activity was evaluated using the parameter of SERS EF, as shown in the following formula: EF = (*I_SERS_* × *C_Raman_*) / (*I_Raman_* × C*_SERS_*). *I*_SERS_ and *I*_Raman_ are the SERS and ordinary Raman intensity at the same Raman shift (1341 cm^-1^), respectively. *C*_Raman_ refers to the concentration of 4-NTP that can produce a Raman signal, and *C*_SERS_ is the final concentration of 4-NTP mixed with plasmonic NPs.

**5.3 Antimicrobial activity evaluation:** In the antibacterial ability test of the AgNPs and ES-AuAgNPs. *Vbrio parahaemolyticus* was enriched in 2216E liquid medium, then centrifuged and washed three times with 1×PBS buffer. The OD_600_ of bacteria was brought into 1, 100 μL of washed bacteria, 100 μL/mL of 100 μg/mL AgNPs or ES-AuAgNPs were subsequently added into 10 mL 2216E liquid medium. Bacterial growth kinetics were monitored via OD_600_ measurements.

In the antibacterial ability test of the control NPs and ES-AuAgNPs. 100 μL OD=1 *Vbrio parahaemolyticus* and 100 μL of different concentrations of plasmonic NPs were subsequently spread plated onto the *parahaemolyticus*-selective agar medium. Following 12 hours of incubation, bacterial counts were conducted.

**5.4 Stability evaluation:** 0.2 nM plasmonic NPs were mixed with varying concentrations of NaCl in a total volume of 250 µL and then kept at room temperature for 2 hours. UV-vis spectroscopy was then used to measure changes in absorbance intensity, determining their maximum tolerable NaCl concentration.

**6 Application of ES-AuAgNPs**

**6.1 Fabrication of sensing nanoprobe:** 1 µL of 10 µM NTP (or 100 µM TP or 1 mM 4-FTP) was added to 1 mL of 0.2 nM ES-AgAuNP, and the mixture was left undisturbed at room temperature for 30 minutes to allow for Raman reporter modification.^10^ Next, thiol-modified DNA aptamers (VP, VV, and VA) were conjugated on the surfaces of 4-NTP, 4-TP, and 4-FTP modified ES AuAgNPs, respectively. For this step, 1 µL of 100 µM aptamer, reduced by TCEP, was added to 1 mL of 0.2 nM ES-AuAgNPs containing 0.1% SDS and 10 mM TE buffer. Subsequently, 100 µL of 2 M NaCl was gradually added to the mixture at a rate of 10 µL per hour. After overnight incubation, the aptamer was successfully conjugated to the surface of the Raman reporter-modified ES-AuAgNPs, forming sensing nanoprobes. Lastly, these sensing nanoprobes were washed twice with water and resuspended in 1 mL of 1×PBS buffer for future use.

**6.2 Analysis procedure:** For screening detection, 400 µL of a sample containing various concentrations of *Vibrio* or other bacteria was mixed with 100 µL of 0.1 nM sensing nanoprobes, and then incubated for 10 minutes at ambient temperature. A 5 mL syringe was taken to draw the reacted mixture, and pass it through an assembled filter set with 5 µm and 0.8 µm filters. The liquid was filtered into a buffer filled with 500 µL of NaAc-HAc buffer containing 1 mg TMB and 1 mol H_2_O_2_. For the classification analysis, we further removed the 0.8 µm filter membrane and employed the Raman imaging to identify *Vibrio* bacteria. The Raman imaging conditions were as follows: the mapping area was set as 1000 x 1000 µm² with a 50 µm step, and the exposure time and laser power were 0.5 seconds and 1 mW, respectively.

**7. REFERENCES**

1. Schwartzberg, A. M.; Olson, T. Y.; Talley, C. E.; Zhang, J. Z. Synthesis, Characterization, and Tunable Optical Properties of Hollow Gold Nanospheres. *J. Phys. Chem. B* **2006**, *110* (40), 19935–19944.
2. Zhang, W.; Duan, H.; Chen, R.; Ma, T.; Zeng, L.; Leng, Y.; Xiong, Y. Effect of Different-Sized Gold Nanoflowers on the Detection Performance of Immunochromatographic Assay for Human Chorionic Gonadotropin Detection. *Talanta* **2019**, *194*, 604–610.
3. Bastús, N. G.; Comenge, J.; Puntes, V. Kinetically Controlled Seeded Growth Synthesis of Citrate-Stabilized Gold Nanoparticles of up to 200 Nm: Size Focusing versus Ostwald Ripening. *Langmuir* **2011**, *27* (17), 11098–11105.
4. Chen, M.; Tang, J.; Luo, W.; Zhang, Z.; Zhu, Y.; Wang, R.; Yang, H.; Chen, X. Core-Shell-Satellite Microspheres-Modified Glass Capillary for Microsampling and Ultrasensitive SERS Spectroscopic Detection of Methotrexate in Serum. *Sens. Actuators B Chem.* **2018**, *275*, 267–276.
5. Zheng, Y.; Zhong, X.; Li, Z.; Xia, Y. Successive, Seed-Mediated Growth for the Synthesis of Single-Crystal Gold Nanospheres with Uniform Diameters Controlled in the Range of 5–150 Nm. *Part. Part. Syst. Charact.* **2014**, *31* (2), 266–273.
6. Choi, N.; Dang, H.; Das, A.; Sim, M. S.; Chung, I. Y.; Choo, J. SERS Biosensors for Ultrasensitive Detection of Multiple Biomarkers Expressed in Cancer Cells. *Biosens. Bioelectron.* **2020**, *164*, 112326.
7. Mdluli, P. S.; Sosibo, N. M.; Mashazi, P. N.; Nyokong, T.; Tshikhudo, R. T.; Skepu, A.; van der Lingen, E. Selective Adsorption of PVP on the Surface of Silver Nanoparticles: A Molecular Dynamics Study. *J. Mol. Struct.* **2011**, *1004* (1), 131–137.
8. Werner, W. S. M.; Glantschnig, K.; Ambrosch-Draxl, C. Optical Constants and Inelastic Electron-Scattering Data for 17 Elemental Metals. *J. Phys. Chem. Ref. Data* **2009**, *38* (4), 1013–1092.
9. Rioux, D.; Vallières, S.; Besner, S.; Muñoz, P.; Mazur, E.; Meunier, M. An Analytic Model for the Dielectric Function of Au, Ag, and Their Alloys. *Adv. Opt. Mater.* **2014**, *2* (2), 176–182.
10. Wu, Y.; Dang, H.; Park, S.-G.; Chen, L.; Choo, J. SERS-PCR Assays of SARS-CoV-2 Target Genes Using Au Nanoparticles-Internalized Au Nanodimple Substrates. *Biosens. Bioelectron.* **2022**, *197*, 113736.

Table S1. Similarities and Differences of Control NPs toward ES-AuAgNPs

| **Name** | **Similarities** | **Differences** |
| --- | --- | --- |
| HAuNPs | Alloyed nanoparticles with a well-accessible surface^*^ | Limited hotspots |
| AuNFs | External hotspots with a well-accessible surface | Mono-metal |
| AuNPs@Ag | Alloyed nanoparticles with a well-accessible surface | Internal hotspots^**^ |
| S-AgAuNPs | Alloyed nanoparticles with external hotspots | Weak accessible ability^***^ |

^*^The HAuNPs, AuNFs, and AuNPs@Ag show a well-accessible surface given the citrate ligand capping.

^**^The hotspots were produced in the internal nanogap between the core-shell structure.

^***^The PEI polymer as the capping agent can cause steric hindrance between S-AgAuNPs and molecules to be adsorbed, resulting in a weak accessible ability of S-AgAuNPs.

Table S2. Information of Aptamer Used in This Work.

| **Name** | **Specificity** | **Base sequence****^*^ (5'- 3')** | **Length** |
| --- | --- | --- | --- |
| Aptamer-VP | *Vibrio parahaemolyticus* | SH-^*^(CH_2_)_12_-ATAAGCATGAATTGACCAACCTAAACTTATTCATTTTCCAGCACCTCTAATATTACTGGC | 60 nt |
| Aptamer-VC | *Vibrio*  *vulnificus* | SH-(CH_2_)_12_-AGTATACGTATTACCTGCAGCCAATCATGACCGCCCACCTCACTCGGCAAGATCTCCGAGATATCG | 66 nt |
| Aptamer-VA | *Vibrio alginolyticus* | SH-(CH_2_)_12_-TCAGTCGCTTCGCCGTCTCCTTCAGCCGGGGTGGTCAGTAGGAGCAGCACAAGAGGGAGCACAAGAGGGAGACCCCAGAGGG | 82 nt |

^*^-(CH_2_)_12_-, used as a spacer to enhance aptamer-protein recognition efficiency


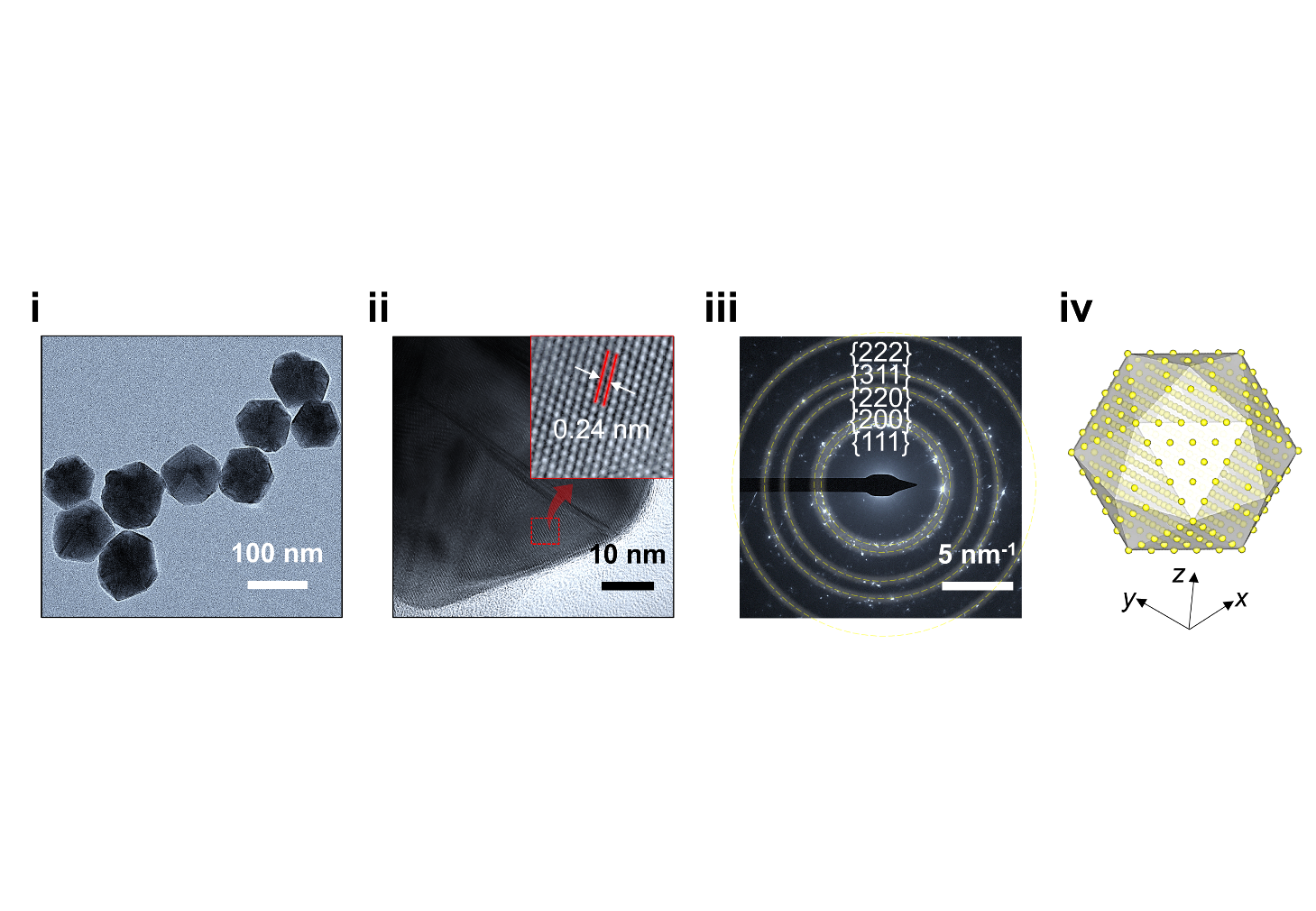


Figure S1. Tannic acid capped-AgNPs of (i) TEM, (ii) HR-TEM image, (iii) SAED patterns, and (iv) morphology visualization by VESTA software.


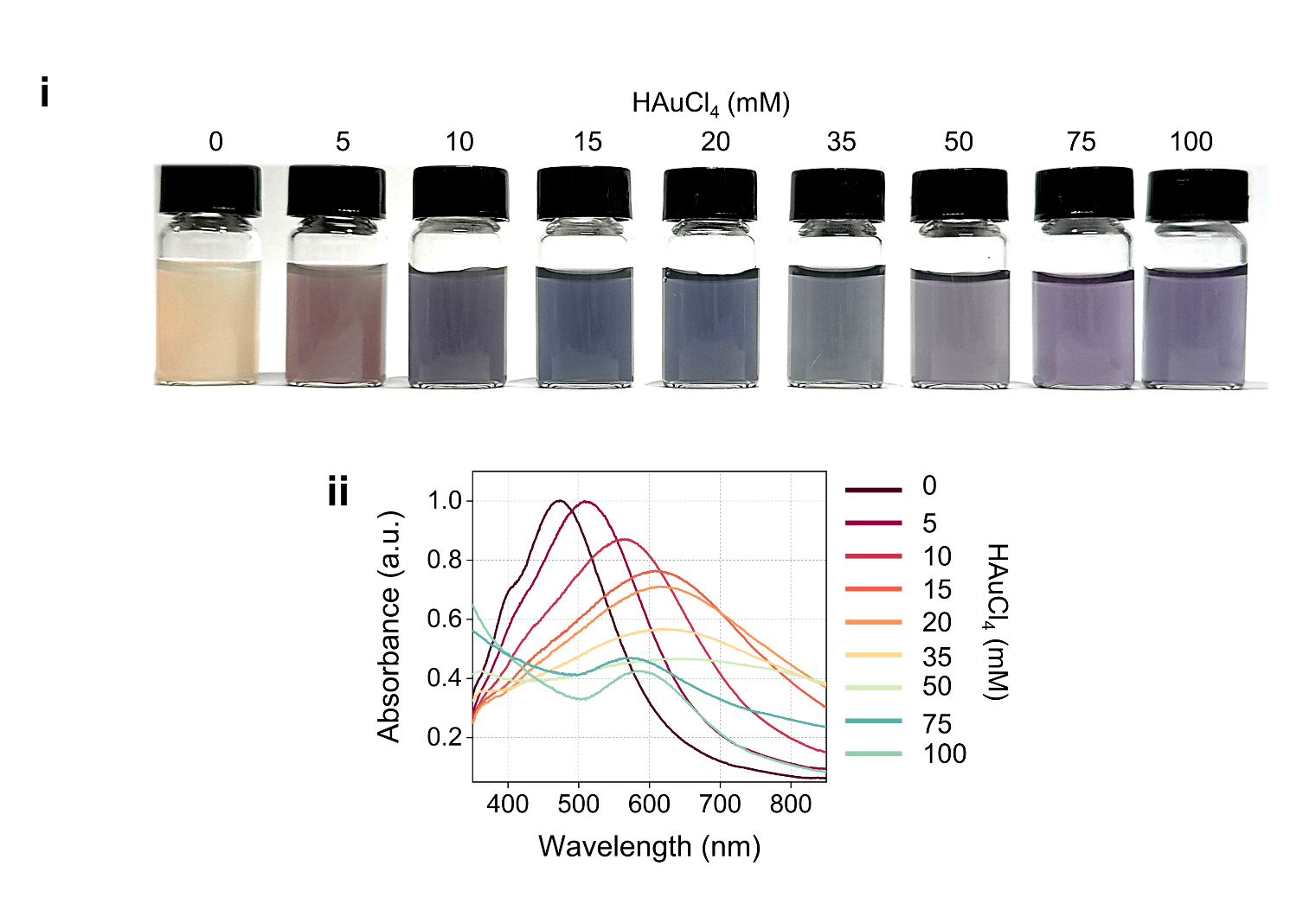


Figure S2. (i) Photography and (ii) UV-vis spectra of ES-AuAgNPs fabricated by different concentrations of HAuCl_4_ etching.


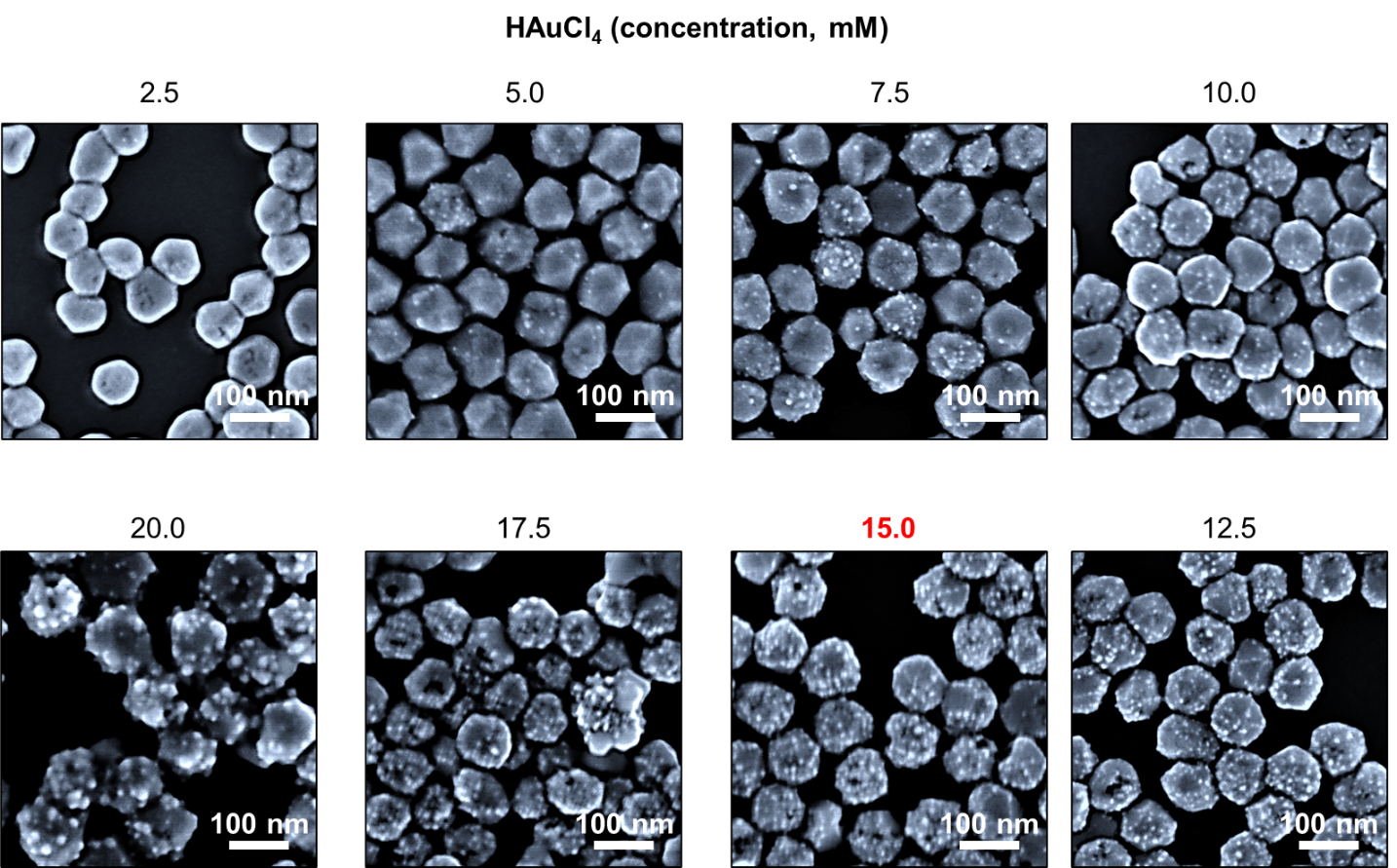


Figure S3. SEM images of ES-AuAgNPs fabricated by different concentrations of HAuCl_4_, from 2.5 mM to 20 mM with 2.5 mM intervals. The optimized concentration of HAuCl_4_ was 15mM.

**
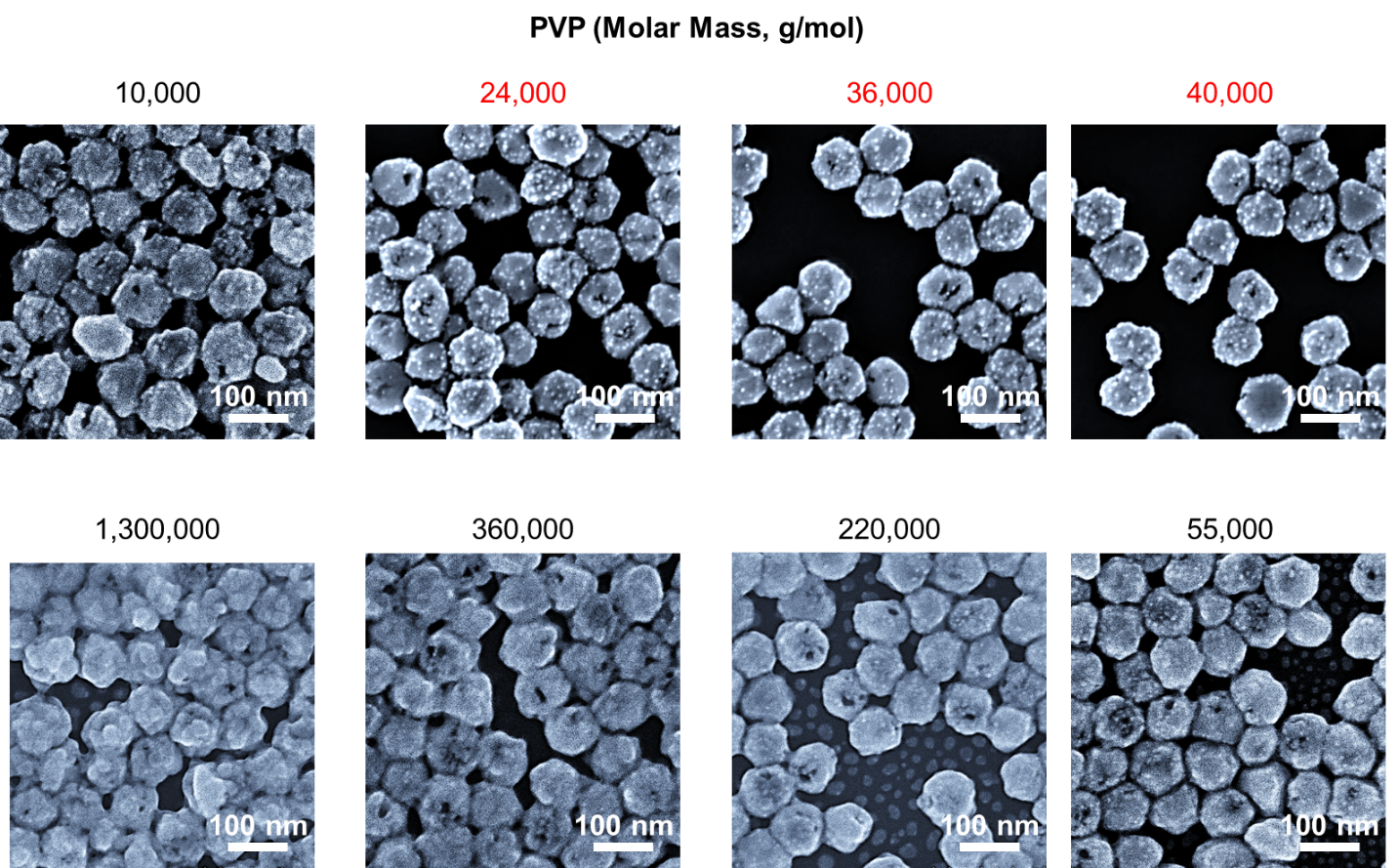
**

Figure S4. SEM images of ES-AuAgNPs fabricated by different molar masses of PVP, from 10,000 to 1,300,000. The optimized molar mass of PVP ranging from 24,000 to 40,000.


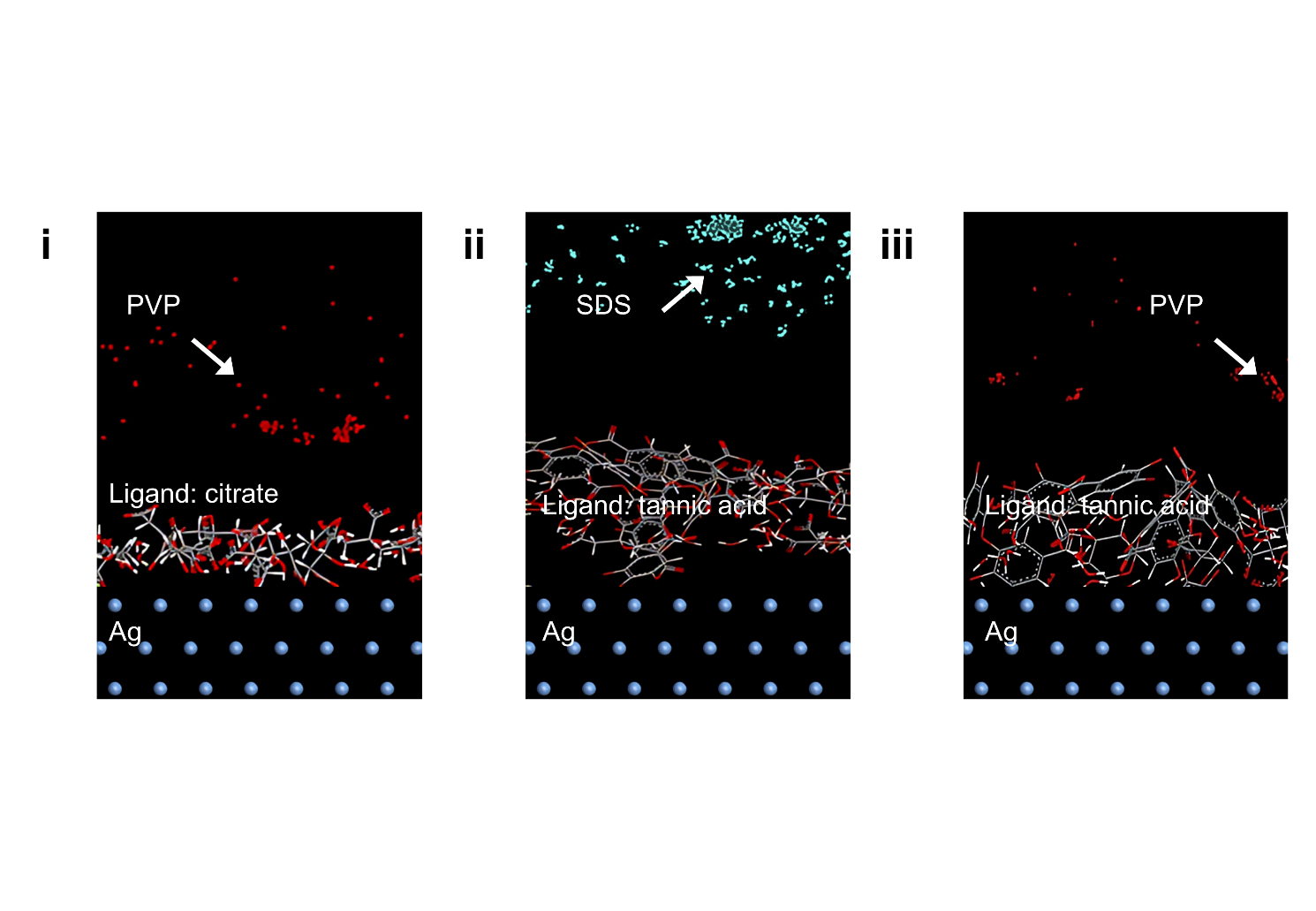


Figure S5. The adsorption behavior of (i) PVP on citrate-AgNPs, (ii) SDS on citrate/tannic acid-AgNPs, and (iii) PVP on citrate/tannic acid-AgNPs. **Compared to SDS, it was observed that PVP molecules moved closer to the surface of Ag, indicating that the binding ability of PVP toward AgNPs is higher than that of SDS.**


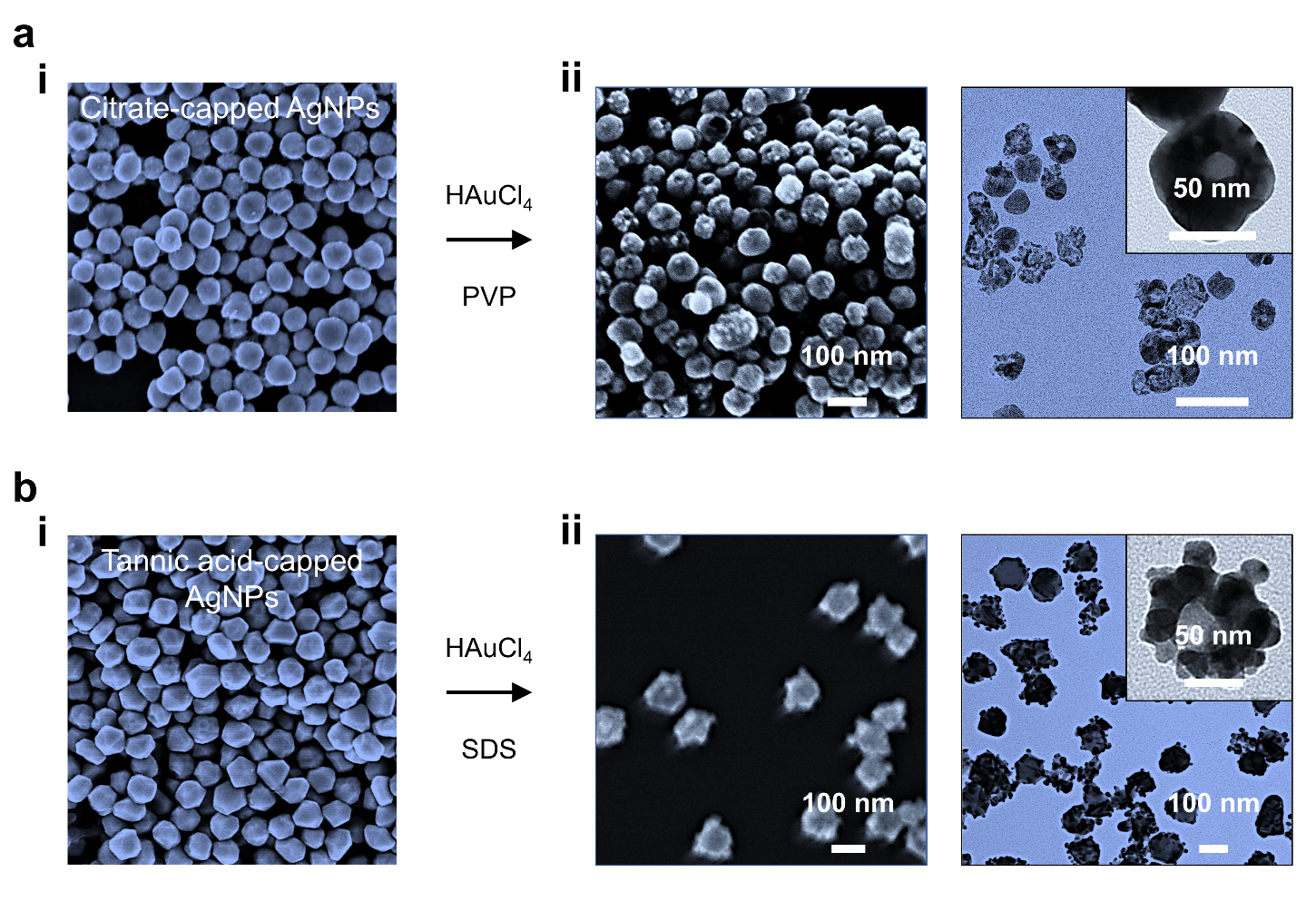


Figure S6. (a) SEM images of (i) citrate capped-AgNPs, and then (ii) treated by PVP and HAuCl_4_. (b) SEM images of (i) tannic acid capped-AgNPs, and then (ii) treated by SDS and HAuCl_4_.


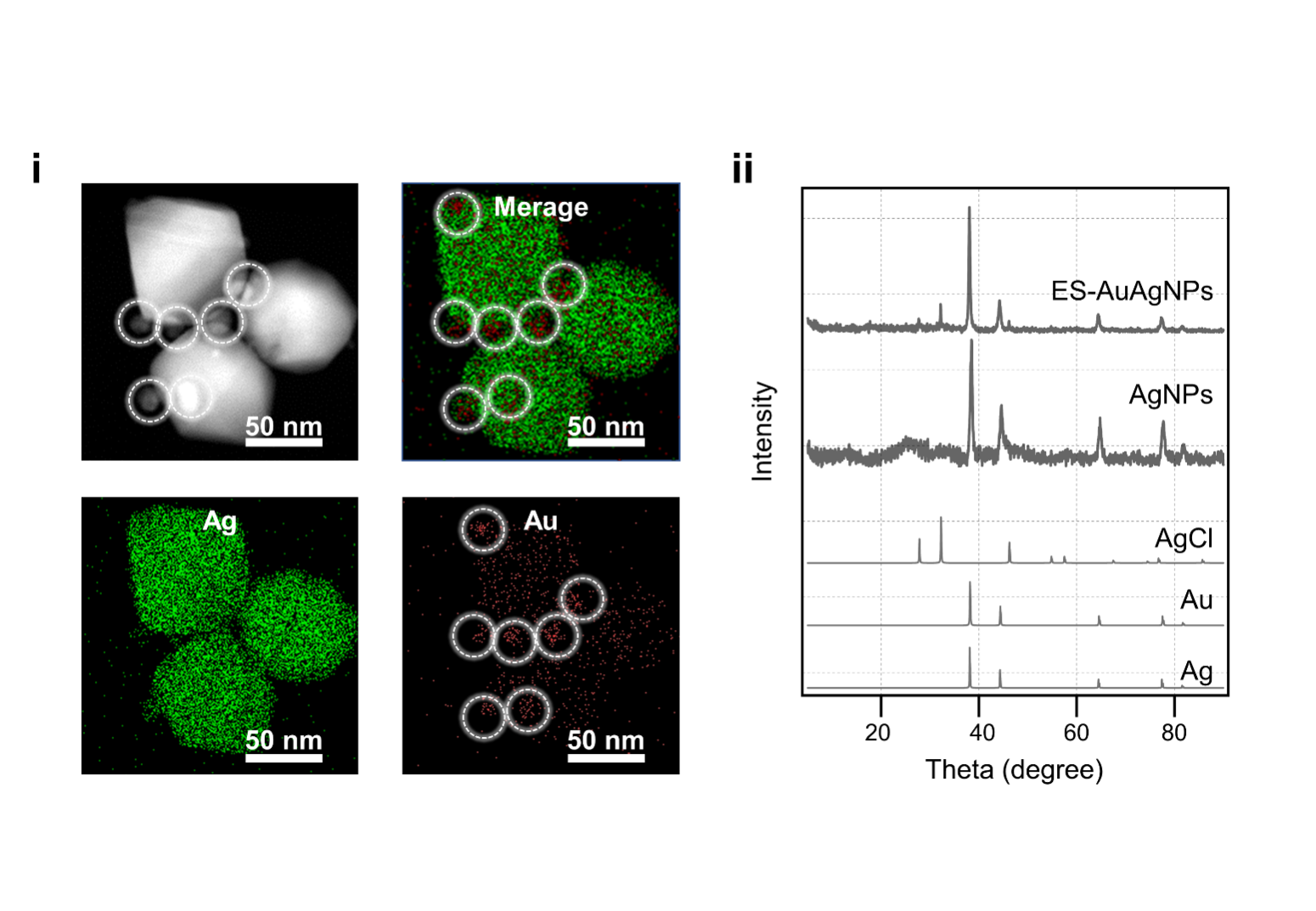


Figure S7. EDS mapping was conducted using ES-AuAgNPs synthesized with a low concentration of HAuCl_4_ (5 mM) instead of an optimized concentration, that is, to reduce background signals of Au and better recognize the characteristic distribution of Au elements. (i) STEM image and EDS mapping profiles of the ES-AuAgNPs fabricated with 5 mM HAuCl_4_. (ii) XRD spectra for ES-AuAgNPs and AgNPs. In the XRD analysis, ES-AuAgNPs retain the primary lattice structure of their AgNPs template, with the main peak corresponding to the {111} lattice. Additionally, the nanoscale AgCl deposition on ES-AgAuNPs enhances various performances, including catalytic activity, antibacterial efficacy, and stability.

**
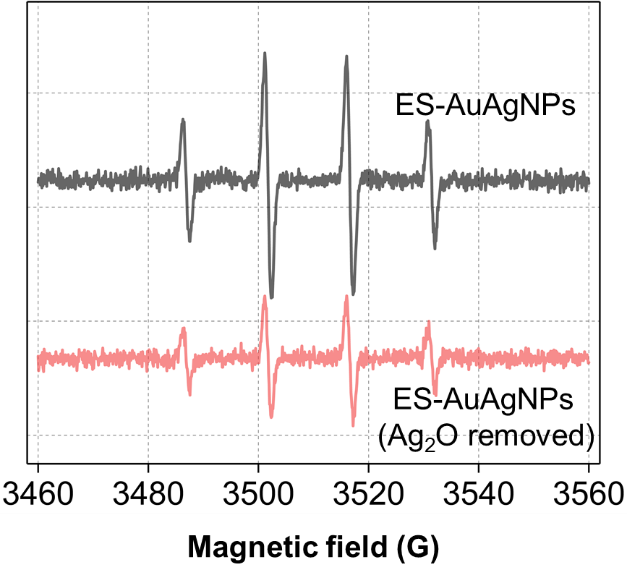
**

Figure S8 EPR spectra of •OH radical for with (black) and without Ag_2_O (red) deposited ES-AuAgNPs.


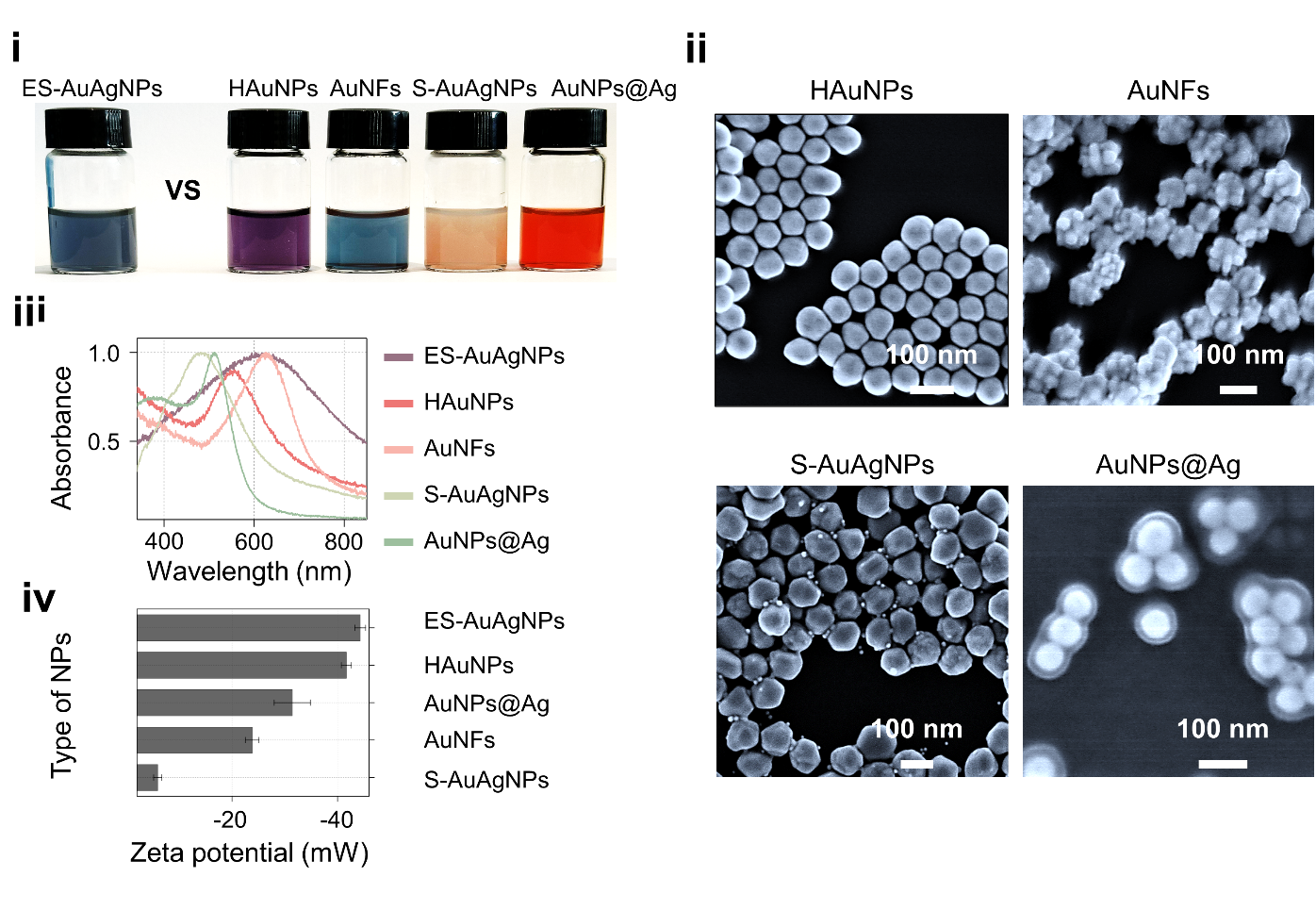


Figure S9. (i) Photography, (ii) SEM images, (iii) UV-vis spectra, and (iv) Zeta potential for ES-AuAgNPs, HAuNP, AuNFs, S-AuAgNPs, and AuNPs@Ag. HAuNPs and ES-AuAgNPs share similar synthesis principles, forming via Au etching on active nanoparticles. Both exhibit comparable surface properties with a well accessibility. However, HAuNPs generate insufficient hotspots. Thus, we used HAuNPs as a control to investigate the impact of hotspots on POD-like catalytic and SERS activity. AuNFs also possess a satellite structure, exhibit sufficient hotspots, and have good surface accessibility, which makes them suitable for assessing the contributions of alloying effects. S-AgAuNPs closely resemble ES-AuAgNPs but form satellite structures through macromolecular surface ligand (PEI)-induced electrostatic adsorption. S-AgAuNPs are used as a control to evaluate how surface accessibility influences multifunctional performance. AuNPs@Ag, similar to ES-AuAgNPs, while generate hotspots within a "core-to-shell" interior structure and suffer from poor stability. We employ AuNPs@Ag to determine whether the openness of hotspots and colloidal stability enhance multifaceted performance.


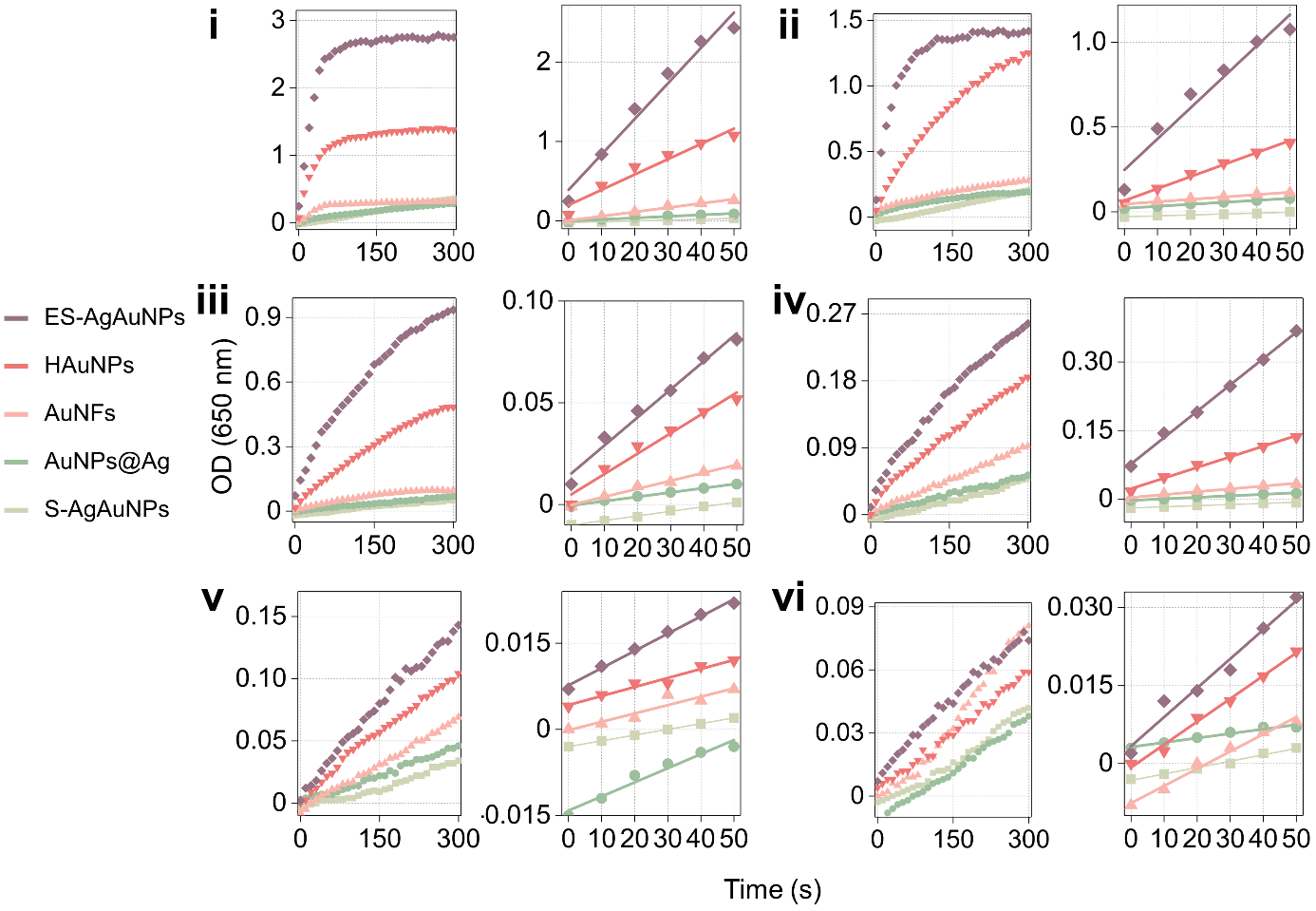


Figure S10. Evaluate the POD-like activity of the five given plasmonic NPs under their various weight. (i) 10 μg, (ii) 5 μg, (iii) 2.5 μg, (iv) 1 μg, (v) 0.5 μg, and (vi) 0.25 μg. Time difference curves (left, 0-300s) and its initial linear portion (right, 0-50s) for the TMB colorimetric reaction, wherein the added TMB and H_2_O_2_ were fixed at 100 μL 10 mg/mL and 100 μL 10 mol/L, respectively.


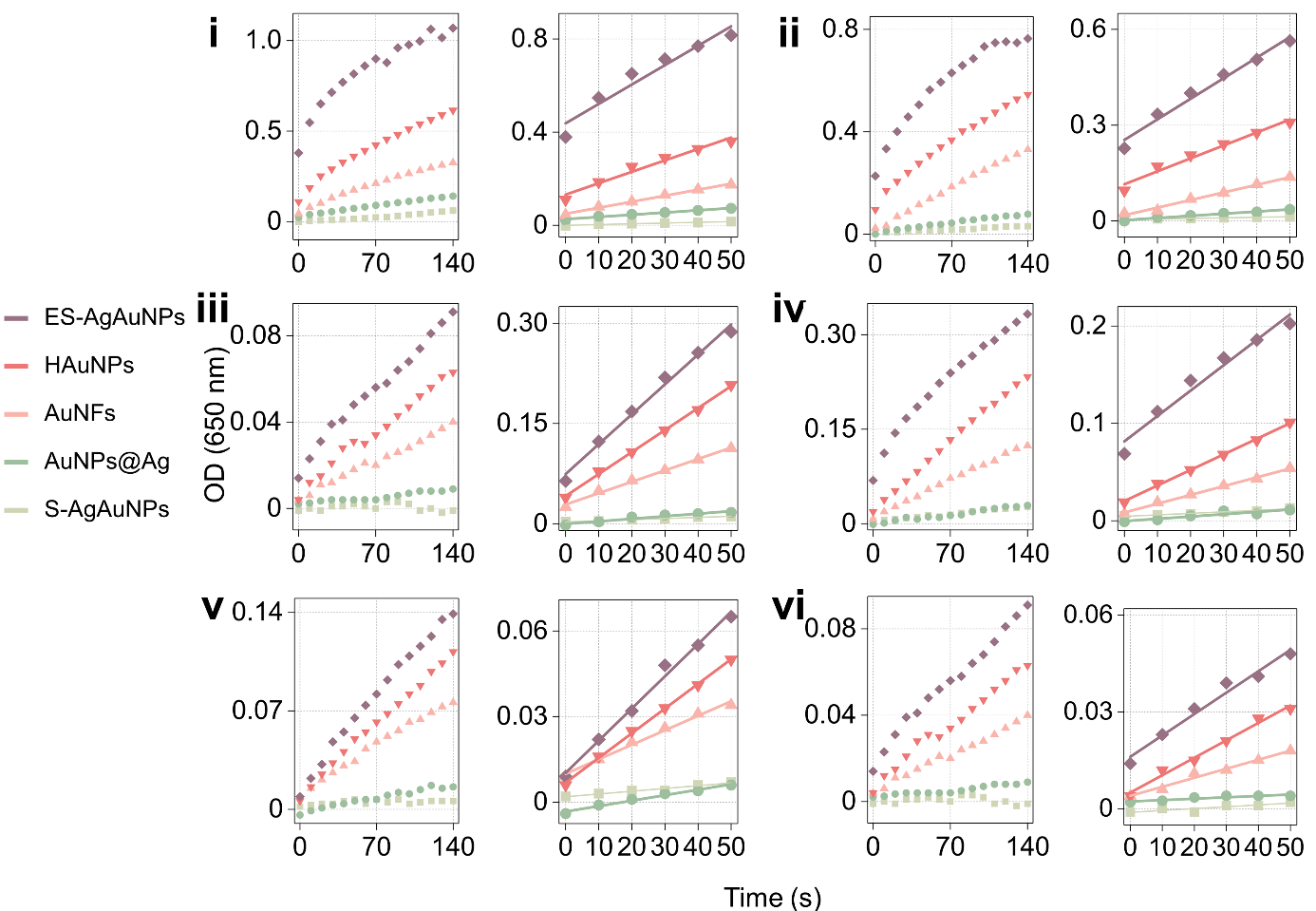


Figure S11. Evaluate the POD-like activity of the five given plasmonic NPs under various H_2_O_2_ concentrations (final concentration). (i) 1 mol/L, (ii) 0.75 mol/L, (iii) 0.5 mol/L, (iv) 0.25 mol/L, (v) 0.1 mol/L, and (vi) 0.05 mol/L. Time difference curves (left, 0-140s) and its initial linear portion (right, 0-50s) for the TMB colorimetric reaction, wherein the TMB and NPs were fixed at 100 μL 10 mg/mL and 100 μL 100 μg/mL, respectively.


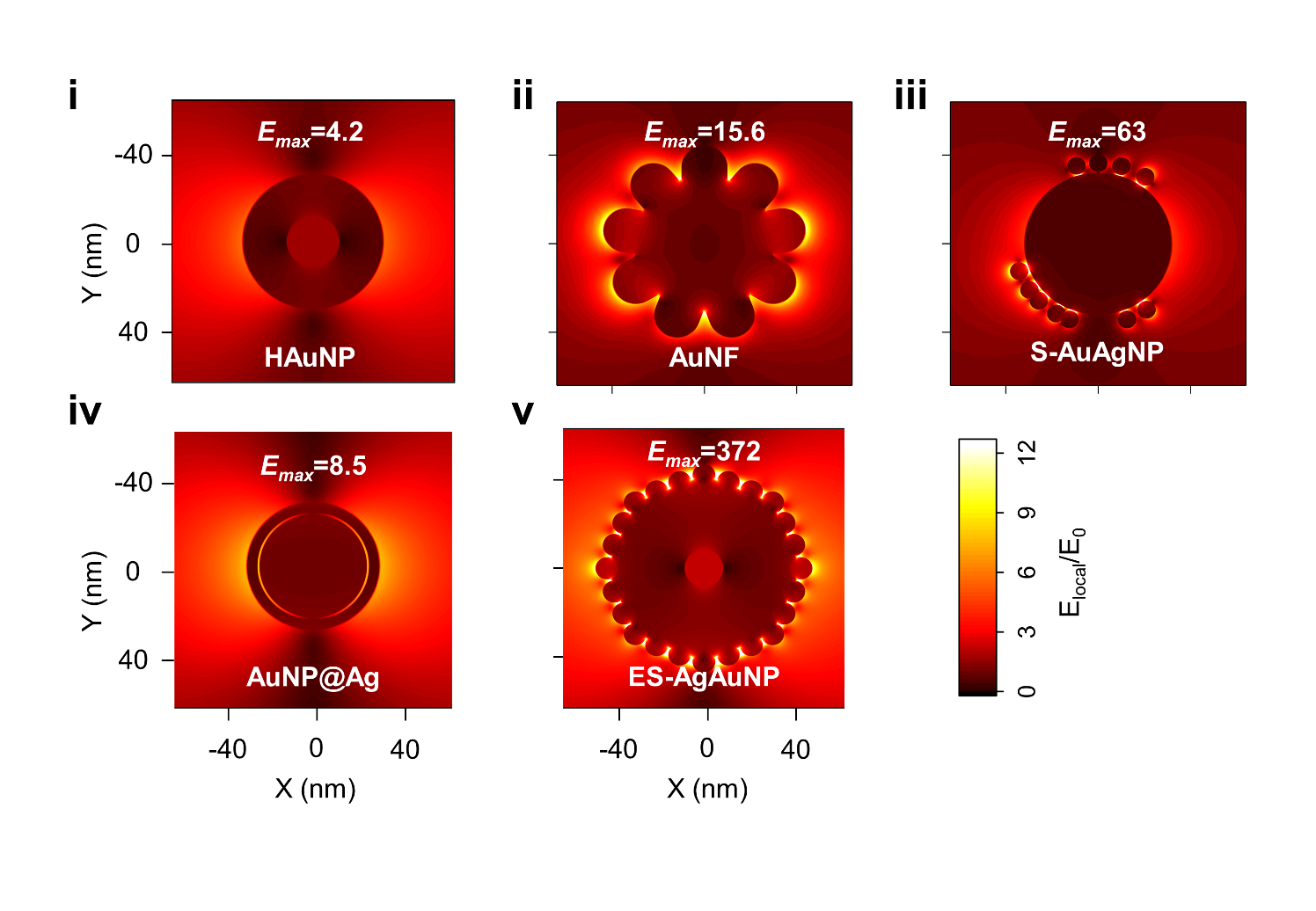


Figure S12. Cross-sectional electric field simulation results of (i) HAuNP, (ii) AuNF, (iii) S-AuAgNP, (iv) AuNP@Ag, and (v) ES-AuAgNP under 633 nm.


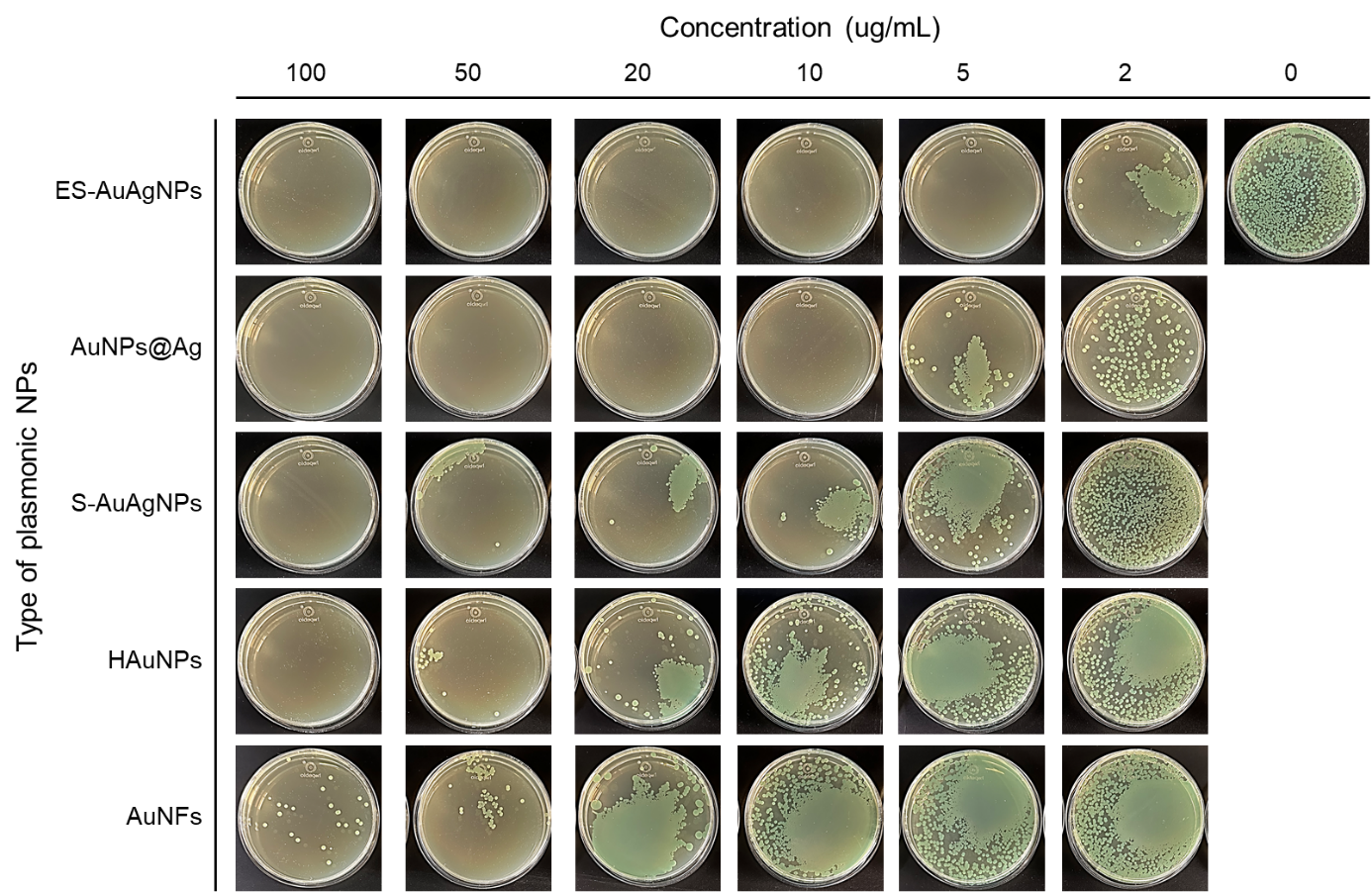


Figure S13. Evaluate the antibacterial activity of the five given plasmonic NPs against *Vibrio parahaemolyticus*. The colony of *Vibrio parahaemolyticus* was incubated with different concentrations of NPs.


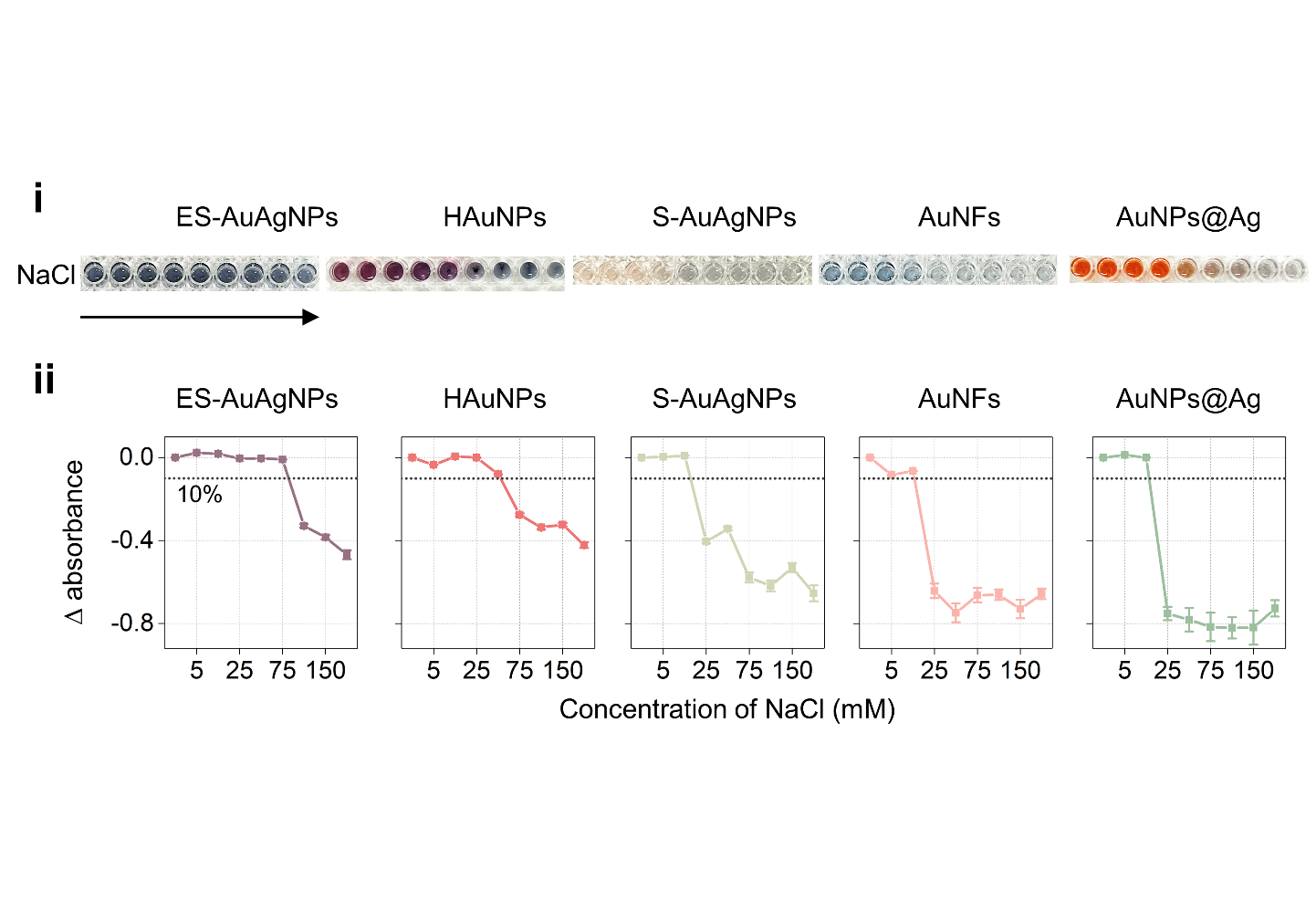


Figure S14. (i) Evaluate the stability of the five given NPs by different concentrations of NaCl incubations, (ii) the maximum NaCl tolerance is defined by an absorbance reduction of no more than 10%.


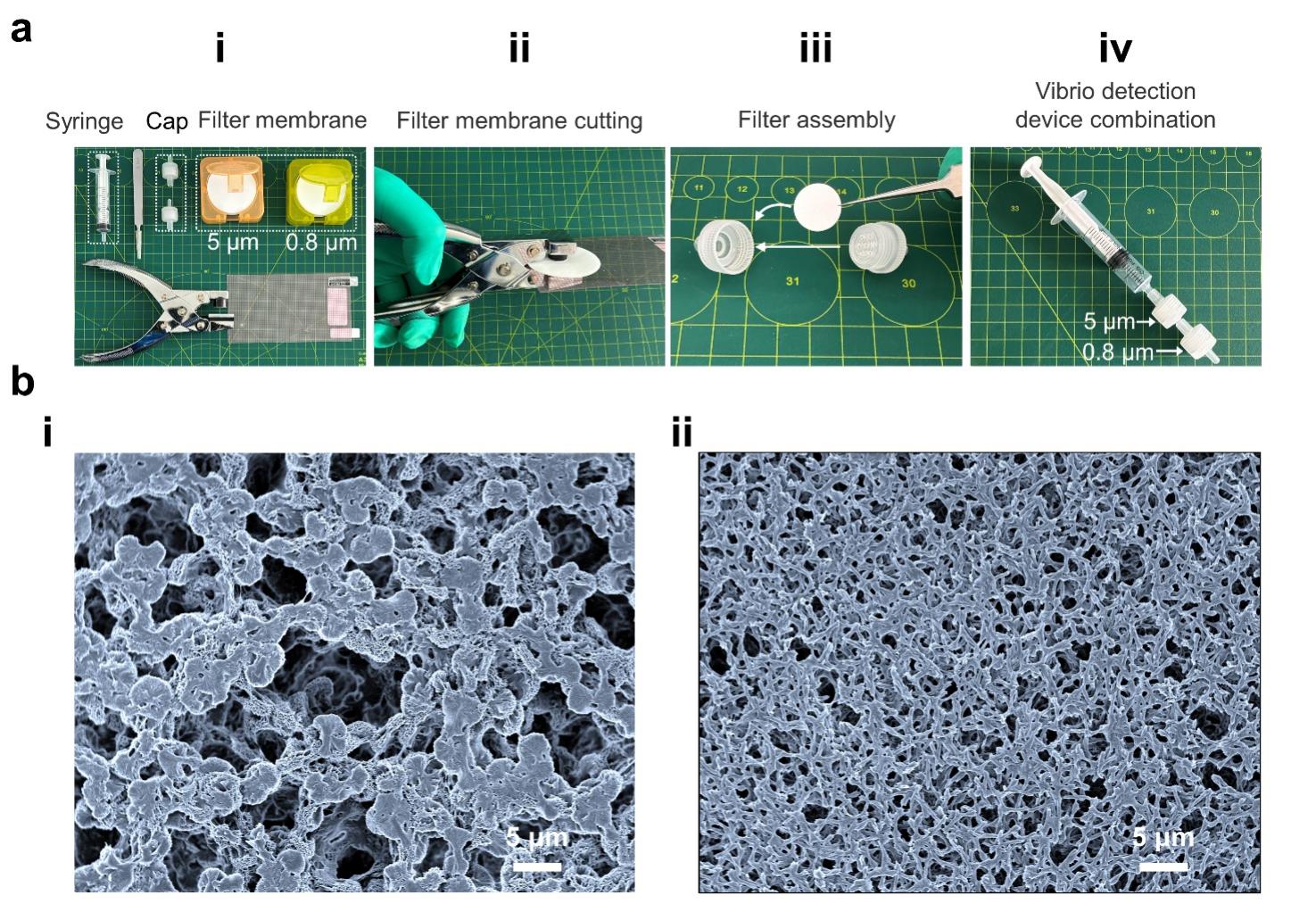


Figure S15 (a) The process for the preparation and installation of the two filters with 5 μm and 0.8 μm pore-sized-membrane. (b) SEM images for the (i) 5 μm and (ii) 0.8 μm pore-sized membranes.


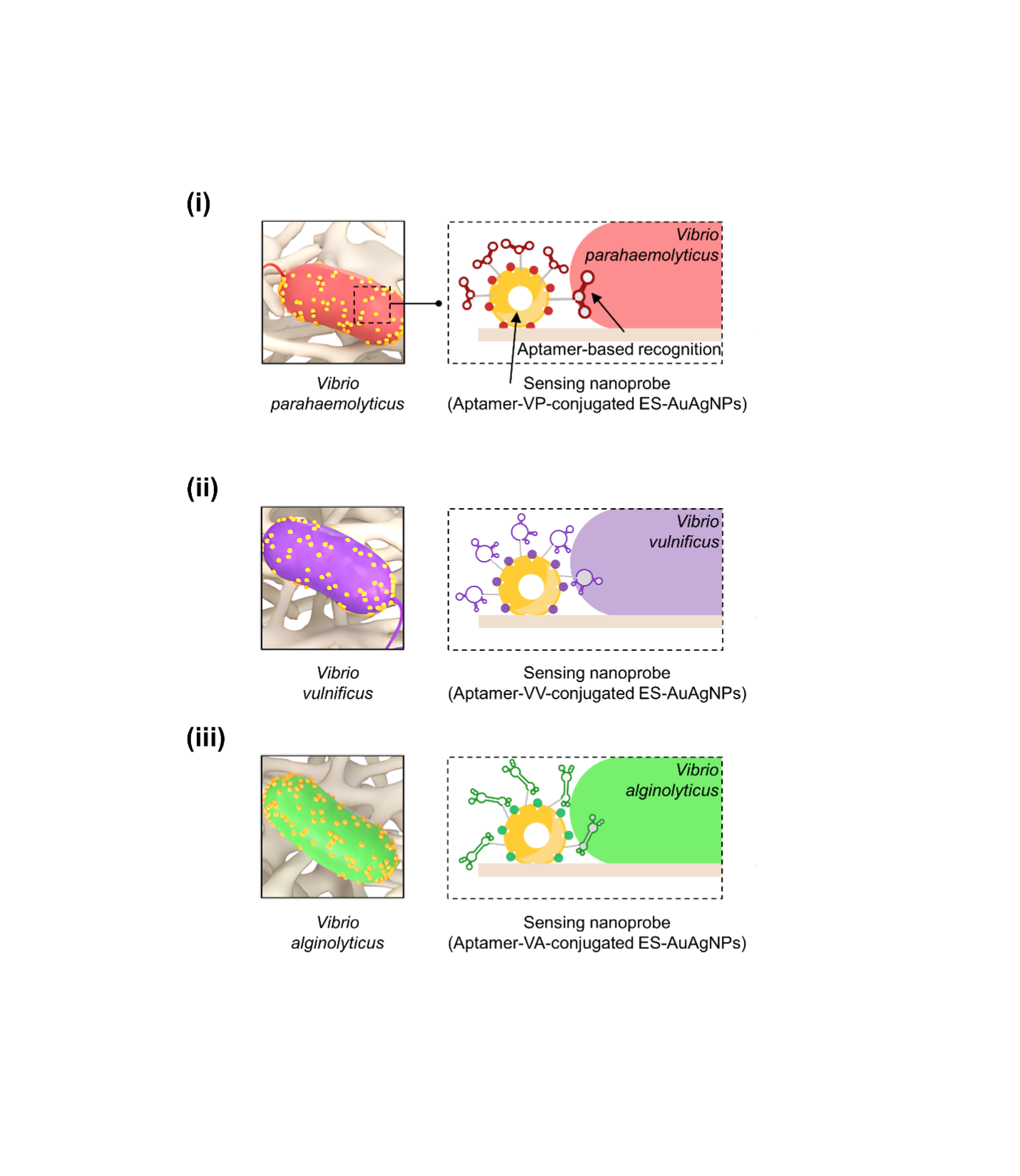


**Figure S16.** (i) The mechanism of sensing nanoprobes specifically recognizes *Vibrio parahaemolyticus*. The sensing nanoprobes were functionalized with an aptamer specific to *Vibrio parahaemolyticus*, enabling them to identify *Vibrio parahaemolyticus* without reacting with other *Vibrio* species. Similarly, (ii) sensing nanoprobe modified with aptamer-VV can specifically recognize only *Vibrio vulnificus*. And, (iii) sensing nanoprobe functionalized with aptamer-VA can identify *Vibrio alginolyticus*.


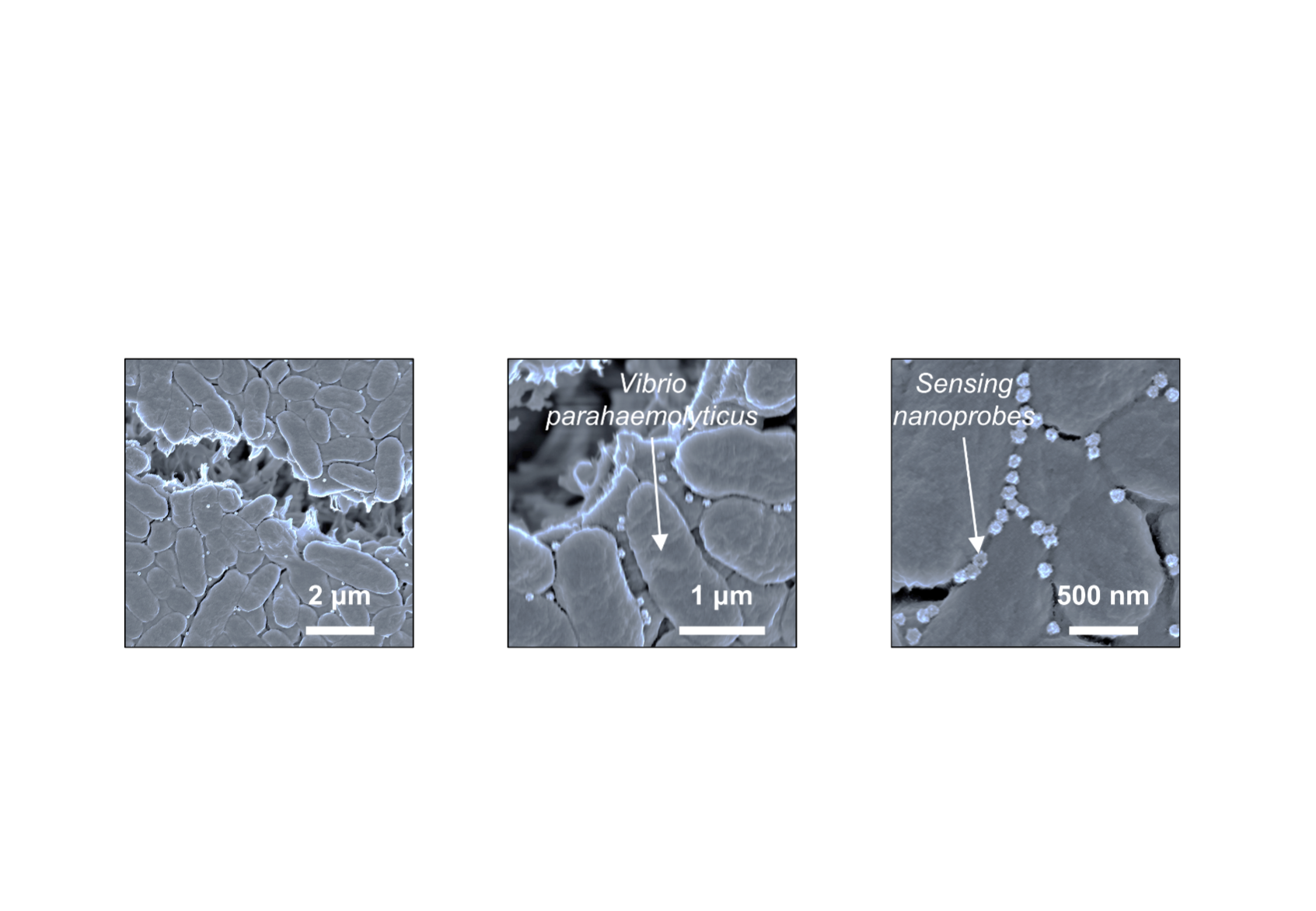


Figure S17. SEM images for the *Vibrio parahaemolyticus* on the 0.8 μm filter membrane's surface.


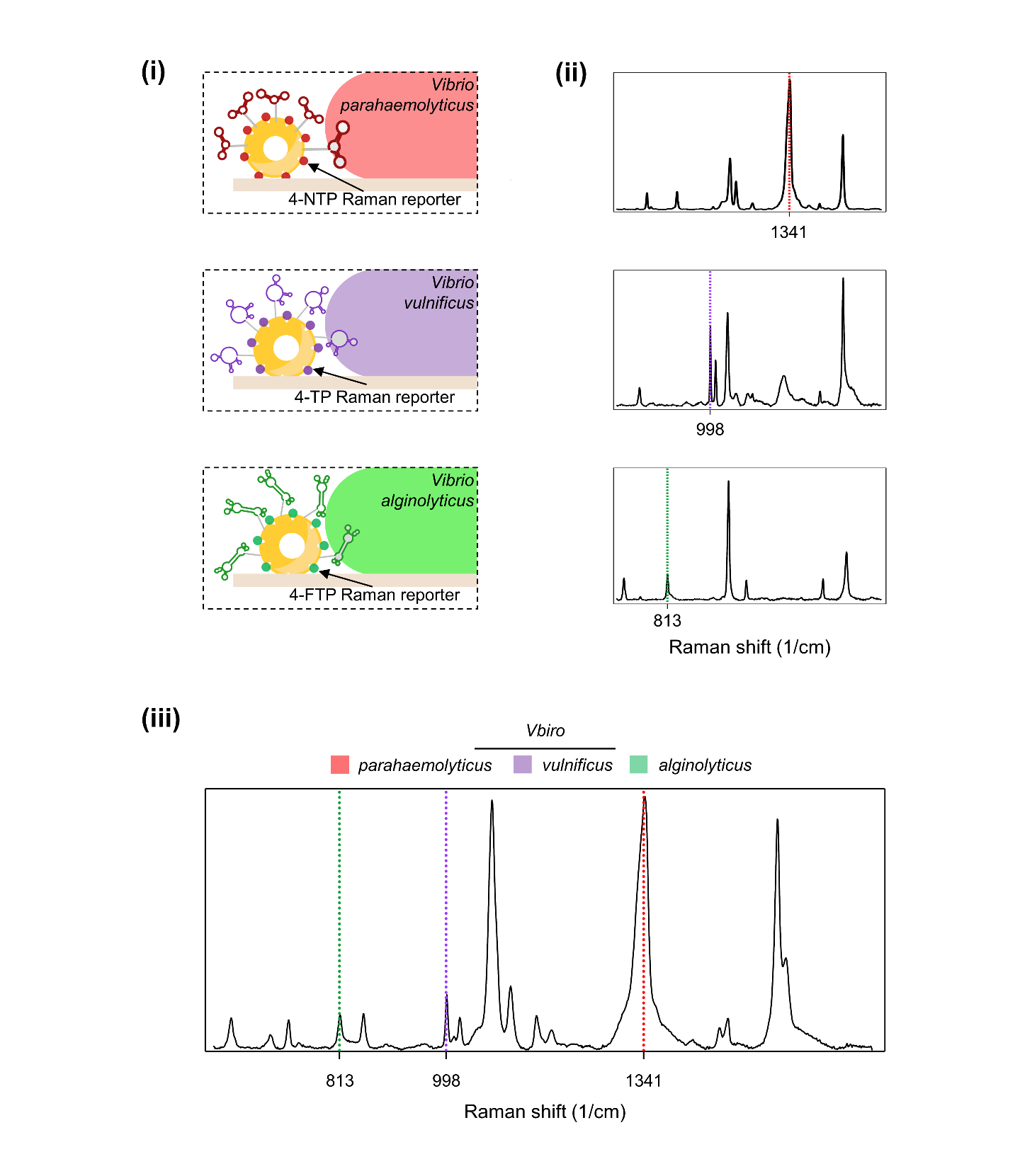


Figure S18. (i) The SERS-based validation mechanism involves using specific sensing nanoprobes against *Vibrio parahaemolyticus*, *Vibrio vulnificus*, and *Vibrio alginolyticus*. These sensing nanoprobes were conjugated with different Raman reporter molecules: 4-NTP for *Vibrio parahaemolyticus*, 4-TP for *Vibrio vulnificus*, and 4-FTP for *Vibrio alginolyticus*. Each of these Raman reporter molecules exhibits distinct Raman fingerprint spectra, allowing validation of the presence of different *Vibrio* bacteria based on their unique fingerprint peaks. Wherein, the Raman spectra and corresponding Raman peak for different combinations of *Vibrio* bacteria: (ii) Individual bacteria – *Vibrio parahaemolyticus*, *Vibrio vulnificus*, and *Vibrio alginolyticus*. (iii) Three bacteria mixed


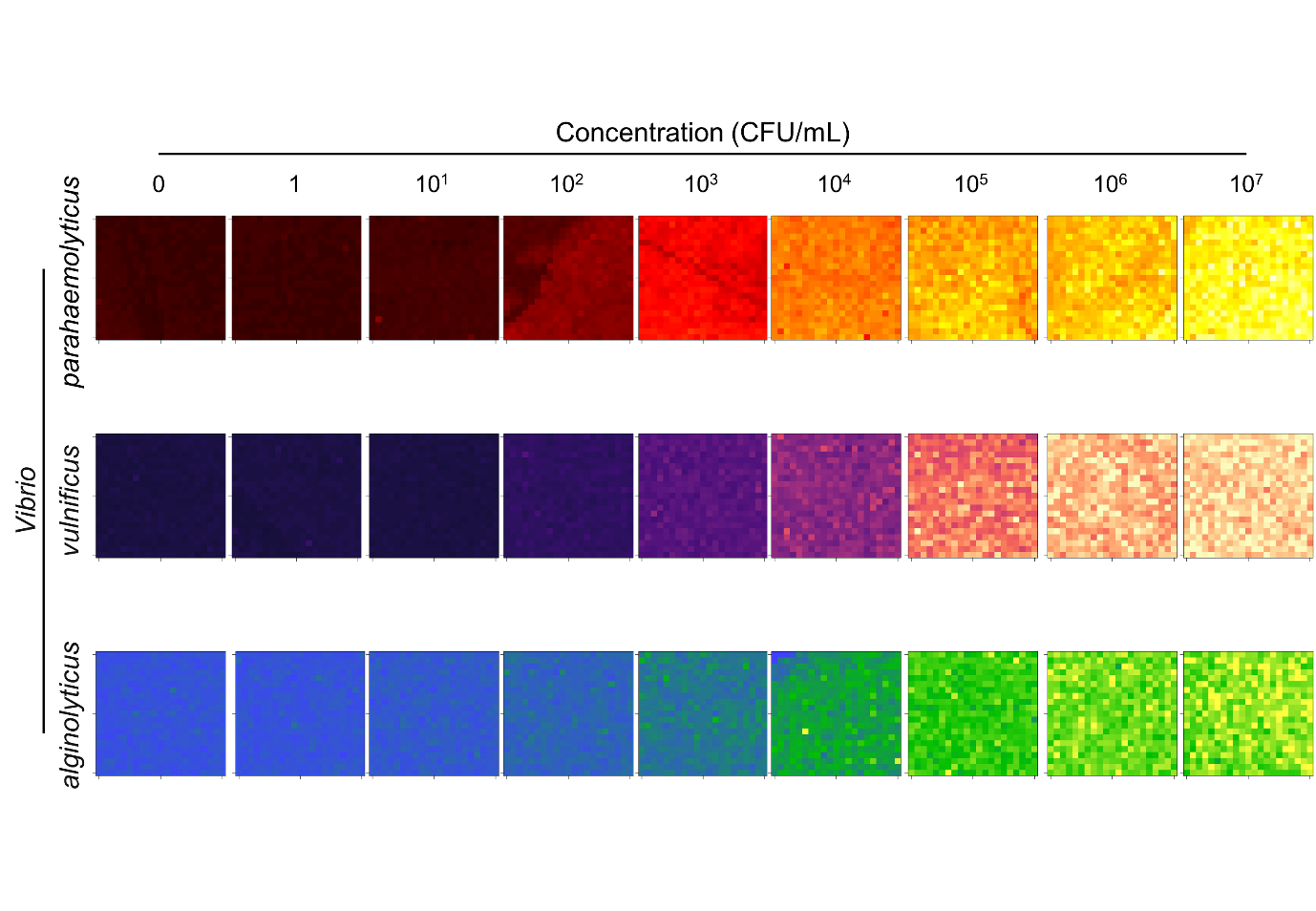


Figure S19. Raman mapping images by scanning an area of 1000×1000 μm^2^ at a 50×50 μm^2^ interval for different concentrations of *Vibrio* bacteria, ranging from 0 to 10^7^ CFU/mL.


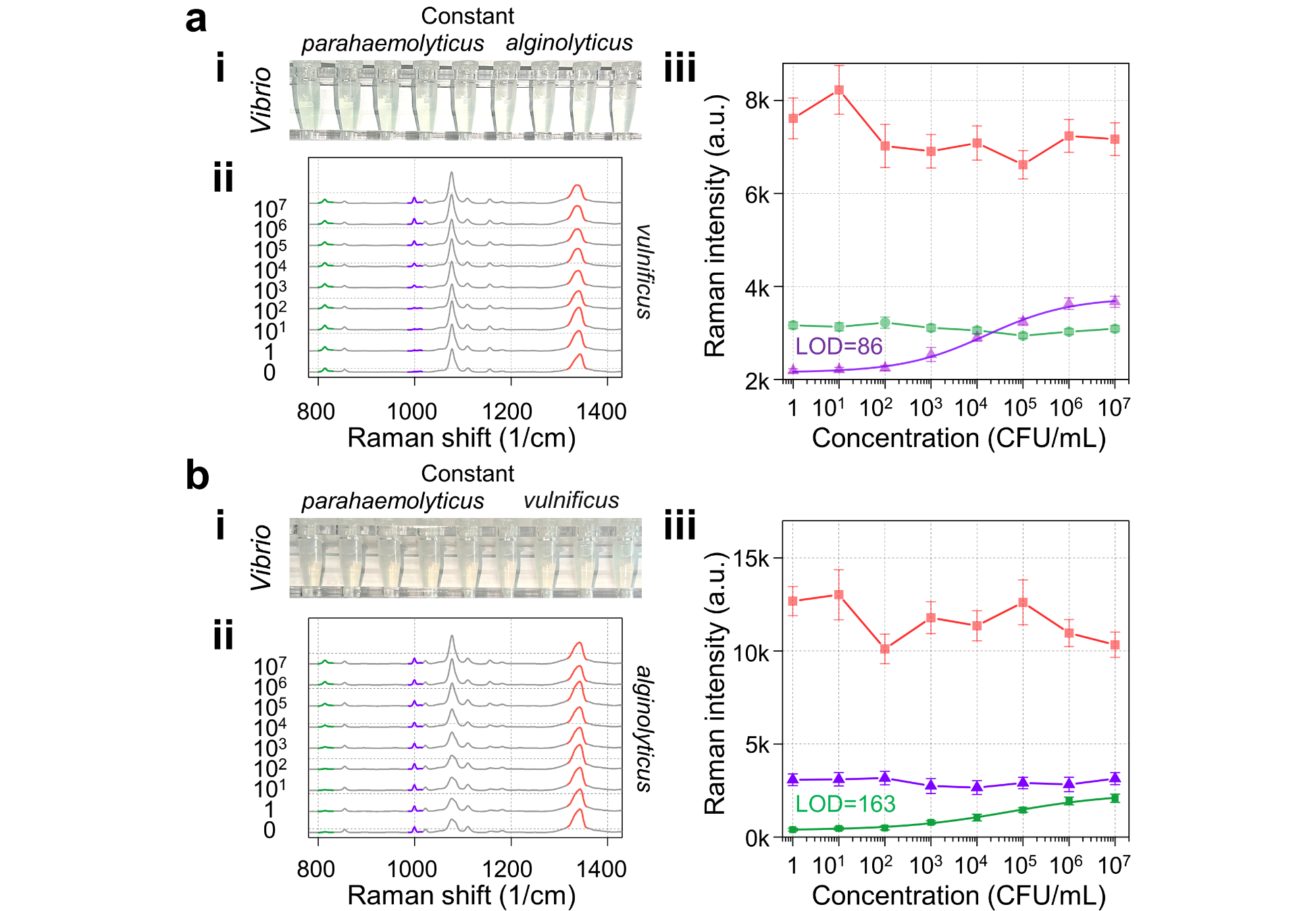


Figure S20. Evaluation of cross-reactivity: (i) the screening photography and (ii) the identification involves Raman spectra with its (iii) intensity, under varied amounts of (a) *Vibrio* *vulnificus* or *Vibrio* *alginolyticus*, (b) along with a fixed amount of the other two *Vibrio* bacteria.


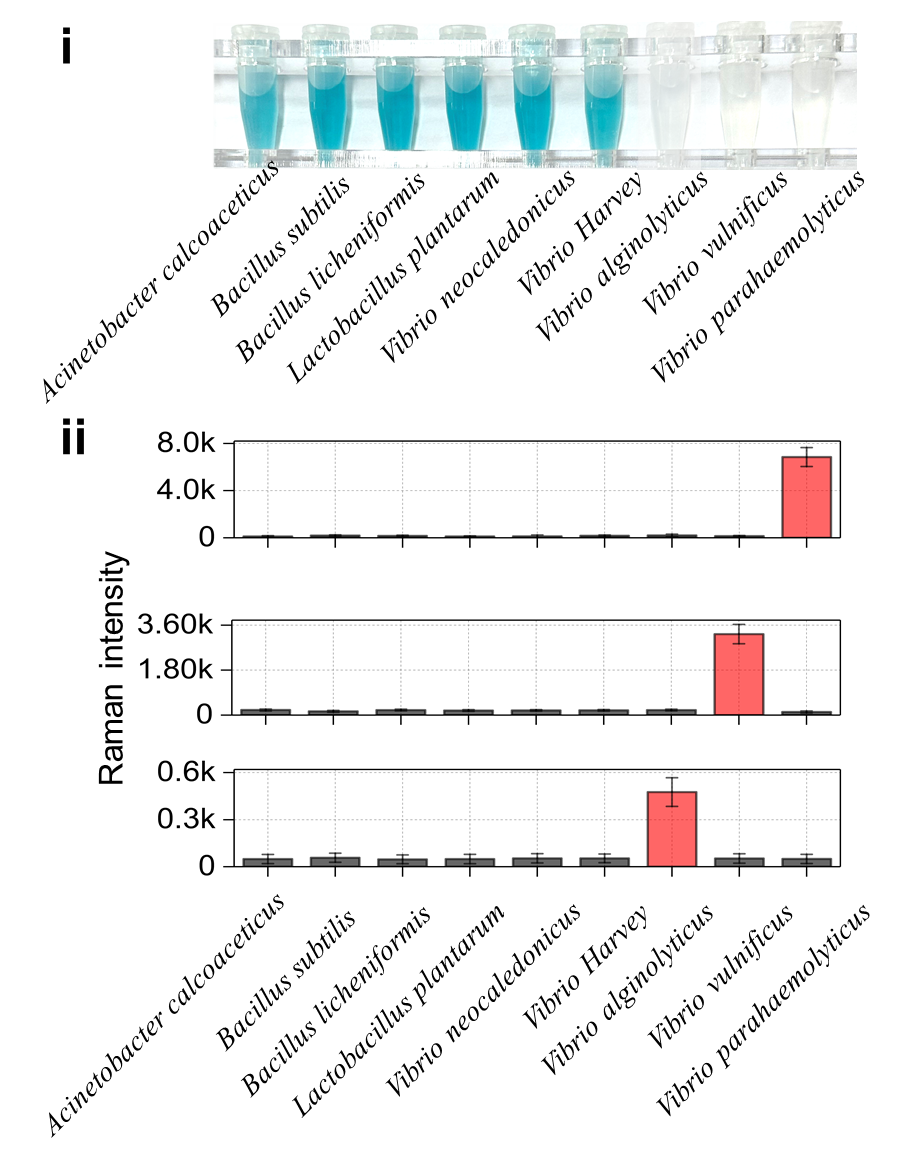


Figure S21. Evaluation of selectivity ability: (i) photography of screening, and (ii) Raman intensity of the identification for various bacteria.


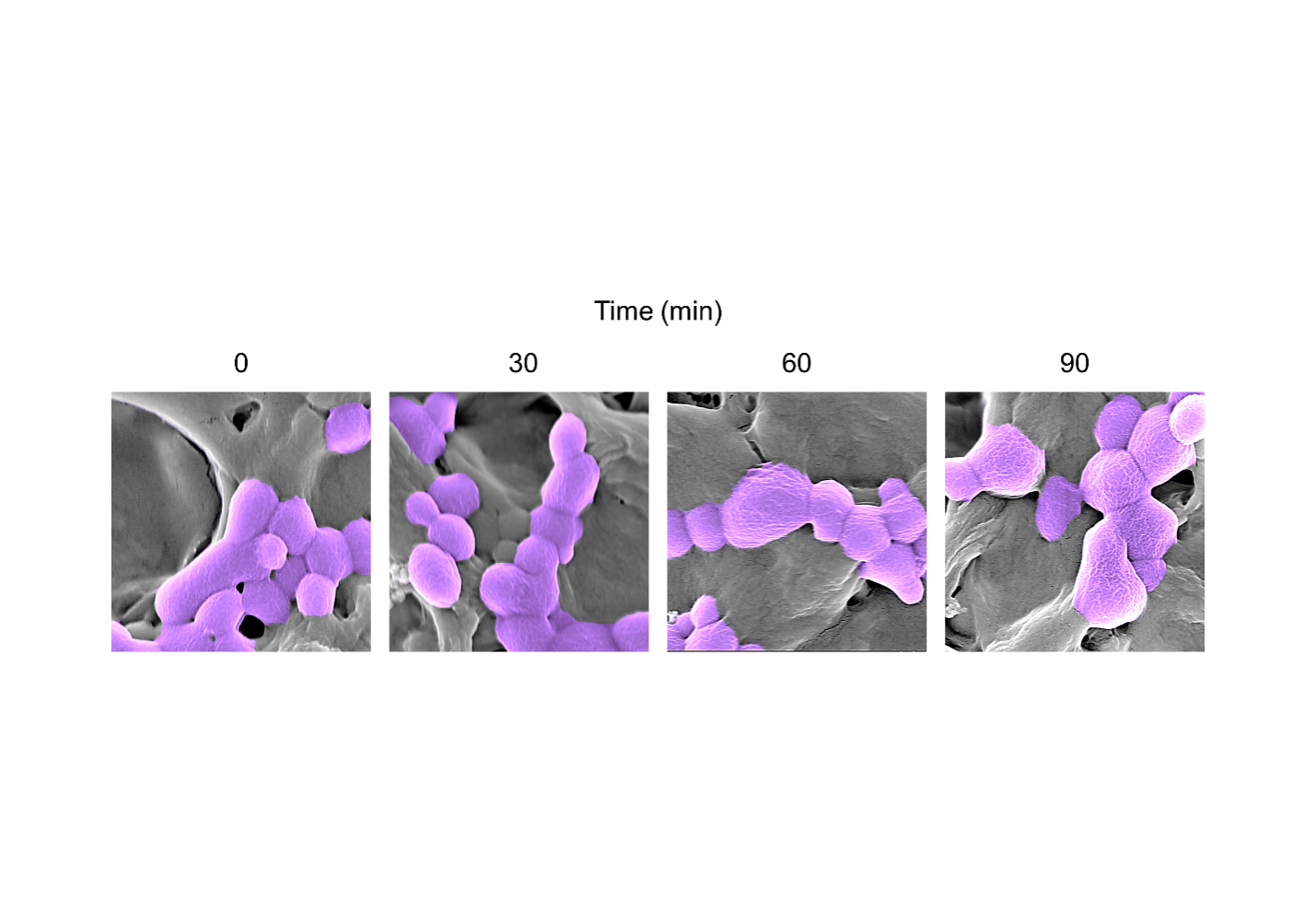


Figure S22. Time difference of SEM images for *Vibrio parahaemolyticus* with 1×PBS buffer treatment
